# Evaluating implementation of the Transparency and Openness Promotion (TOP) guidelines: the TRUST process for rating journal policies, procedures, and practices

**DOI:** 10.1186/s41073-021-00112-8

**Published:** 2021-06-02

**Authors:** Evan Mayo-Wilson, Sean Grant, Lauren Supplee, Sina Kianersi, Afsah Amin, Alex DeHaven, David Mellor

**Affiliations:** 1 Indiana University School of Public Health-Bloomington, Bloomington, IN USA; 2 Indiana University Richard M. Fairbanks School of Public Health, Indianapolis, IN USA; 3 Child Trends, Bethesda, MD USA; 4 Center for Open Science, Charlottesville, VA USA

**Keywords:** Reproducibility, Research transparency, Open science, TOP factor, TOP guidelines

## Abstract

**Background:**

The Transparency and Openness Promotion (TOP) Guidelines describe modular standards that journals can adopt to promote open science. The TOP Factor is a metric to describe the extent to which journals have adopted the TOP Guidelines in their policies. Systematic methods and rating instruments are needed to calculate the TOP Factor. Moreover, implementation of these open science policies depends on journal procedures and practices, for which TOP provides no standards or rating instruments.

**Methods:**

We describe a process for assessing journal policies, procedures, and practices according to the TOP Guidelines. We developed this process as part of the Transparency of Research Underpinning Social Intervention Tiers (TRUST) Initiative to advance open science in the social intervention research ecosystem. We also provide new instruments for rating journal instructions to authors (policies), manuscript submission systems (procedures), and published articles (practices) according to standards in the TOP Guidelines. In addition, we describe how to determine the TOP Factor score for a journal, calculate reliability of journal ratings, and assess coherence among a journal’s policies, procedures, and practices. As a demonstration of this process, we describe a protocol for studying approximately 345 influential journals that have published research used to inform evidence-based policy.

**Discussion:**

The TRUST Process includes systematic methods and rating instruments for assessing and facilitating implementation of the TOP Guidelines by journals across disciplines. Our study of journals publishing influential social intervention research will provide a comprehensive account of whether these journals have policies, procedures, and practices that are consistent with standards for open science and thereby facilitate the publication of trustworthy findings to inform evidence-based policy. Through this demonstration, we expect to identify ways to refine the TOP Guidelines and the TOP Factor. Refinements could include: improving templates for adoption in journal instructions to authors, manuscript submission systems, and published articles; revising explanatory guidance intended to enhance the use, understanding, and dissemination of the TOP Guidelines; and clarifying the distinctions among different levels of implementation.

Research materials are available on the Open Science Framework: https://osf.io/txyr3/.

**Supplementary Information:**

The online version contains supplementary material available at 10.1186/s41073-021-00112-8.

## Background

Research transparency and openness can speed scientific progress and increase trust in science [1]. Evidence that much empirical research cannot be reproduced [2]—including basic experiments in psychology [3], economics [4], and the social sciences [5]—has led to concerns about responsible research conduct and a “reproducibility crisis” [6], with a consequent “credibility revolution” focused largely on increasing research transparency and openness [7].

Published in 2015, the Transparency and Openness Promotion (TOP) Guidelines introduced eight modular standards for transparency and openness: citation standards, data transparency, analytic methods (code) transparency, research materials transparency, design and analysis transparency, study preregistration, analysis plan preregistration, and replication [8]. Using these standards, scientific journals can require that authors disclose whether they used an open science practice (Level 1), require that authors actually use an open science practice (Level 2), or verify themselves that authors used an open science practice according to explicit standards (Level 3). At the time of writing, the TOP Guidelines have over 5000 signatories, and 1100 journals have agreed to implement one or more of these standards, most often by specifying data citation standards (see https://osf.io/2sk9f/ for a list of such journals).

**Fig. 1:**
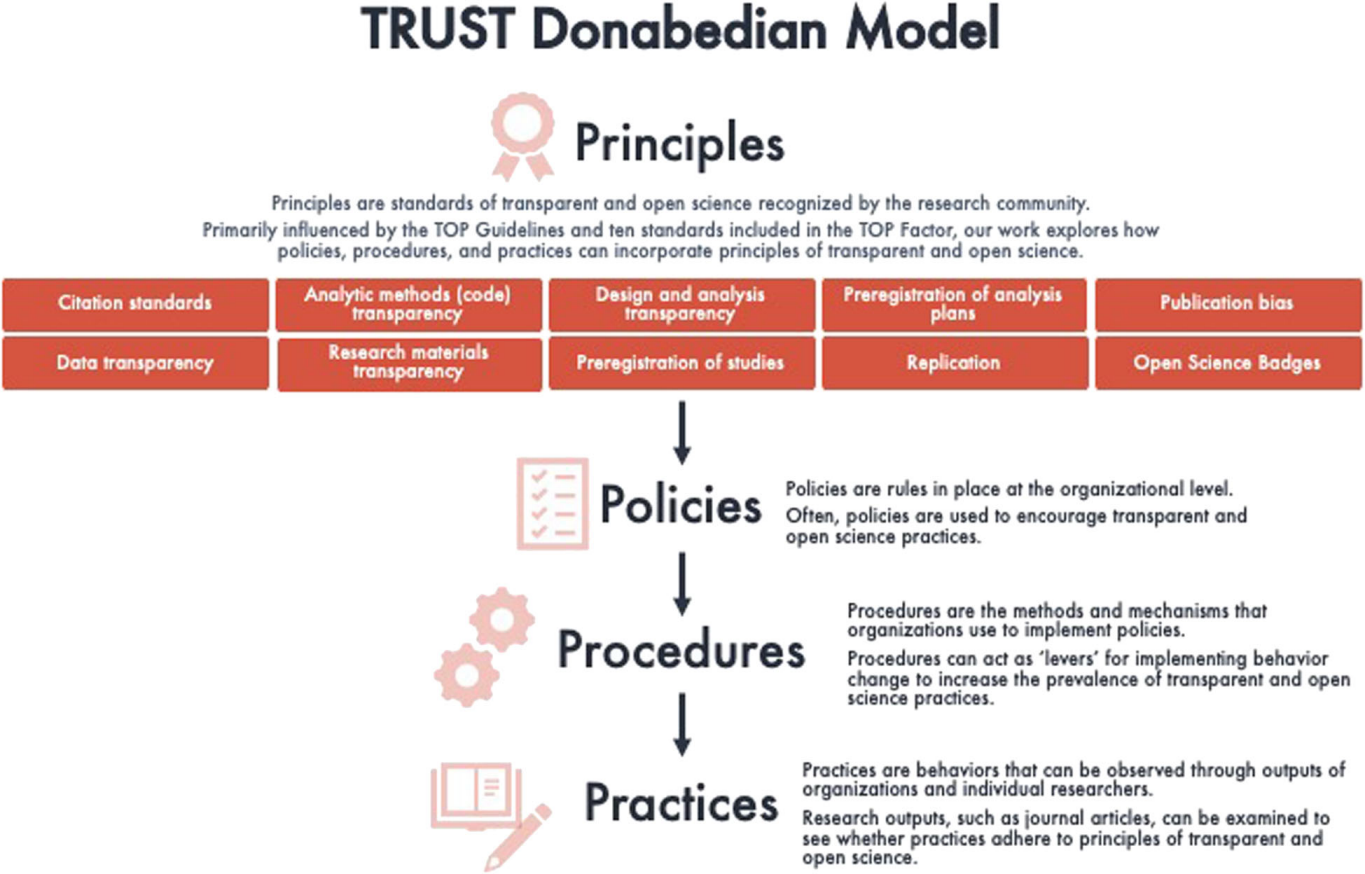
A “Structure-Process-Outcome” Model of the TOP Guidelines

Meta-scientists can use the modular standards in the TOP Guidelines to evaluate whether journals promote transparency and openness by assessing journal structure, process, and outcomes (see Fig. 1). Following public health models for evaluating organizational quality in promoting health [9], the standards in TOP can be conceptualized as principles of transparency and openness that journals operationalize through policies (i.e., instructions to authors and related documents), procedures (i.e., journal submission systems), and practices (i.e., published journal articles).

To assess journal policies, the Center for Open Science (COS) created the “TOP Factor” in 2020. Designed as an alternative to the Journal Impact Factor for evaluating journal quality, the TOP Factor is a quantitative metric that assesses the degree to which journal policies promote transparency and openness. The TOP Factor is calculated as the sum of: journal implementation of the eight modular standards within the TOP Guidelines; an additional standard related to publication bias of original studies (rather than replications); and adoption of open science badges (see Table 1). Several studies already have investigated the TOP Factor of journals within specific disciplines [10, 11]. However, there are no established instruments or processes for calculating the TOP Factor, so journal policies have been rated using bespoke methods or expert judgement. Consequently, the inter-rater reliability of TOP Factor ratings is unknown. Anecdotal evidence suggests that differences in the interpretation and rating of journal policies are common. Crowdsourcing efforts to rate journals according to the TOP Factor have not used methodologically reproducible rating procedures.

**Table 1:** TOP Factor Rubric (https://osf.io/t2yu5/)

Standard	Level 1	Level 2	Level 3
**Data citation**	Journal describes citation of data in guidelines to authors with clear rules and examples.	Article requires appropriate citation for data used consistent with the journal’s author guidelines.	Article is not published until providing appropriate citation for data following journal’s author guidelines.
**Data transparency**	Articles must state whether or not data are available.	Articles must have publicly available data, or explain why ethical/legal constraints prevent it.	Articles must have publicly available data and must be used to computationally reproduce or confirm results prior to publication.
**Analytical code transparency**	Articles must state whether or not code is available.	Articles must have publicly available code, or explain why ethical/legal constraints prevent it.	Articles must have publicly available code and must be used to computationally reproduce or confirm results prior to publication.
**Materials transparency**	Articles must state whether or not materials are available.	Articles must have publicly available materials, or explain why ethical/legal constraints prevent it.	Articles must have publicly available materials and must be used to computationally reproduce or confirm results prior to publication.
**Reporting guidelines**	Journal articulates design transparency standards.	Journal requires adherence to design transparency standards for review and publication.	Journal requires and enforces adherence to design transparency standards for review and publication.
**Study preregistration**	Articles will state if work was preregistered.	Article states whether work was preregistered and, if so, journal verifies adherence to preregistered plan.	Journal requires that confirmatory or inferential research must be preregistered.
**Analysis plan preregistration**	Articles will state if work was preregistered with an analysis plan.	Article states whether work was preregistered with an analysis plan and, if so, journal verifies adherence to preregistered plan.	Journal requires that confirmatory or inferential research must be preregistered with an analysis plan.
**Replication**	Journal encourages submission of replication studies.	Journal will review replication studies blinded to results.	Registered Reports for replications as a regular submission option.
**Publication bias**	Journal states that significance or novelty are not criteria for publication decisions.	Journal will review (novel) studies blinded to results.	Journal accepts Registered Reports for novel studies as a regular submission option.
**Open science badges**	Journal awards 1 or 2 open science badges	Journal awards all 3 open science badges	

In addition to policies described in the TOP Guidelines, journals’ manuscript submission procedures could promote transparency and openness by encouraging or requiring certain practices. That is, many journals require that authors, peer reviewers, and editors handle manuscripts using electronic systems such as Editorial Manager and ScholarOne. Such systems can implement certain policies automatically. For example, electronic systems can require that abstracts be entered in textboxes with word limits. To promote transparency and openness, journals might recommend or require that authors enter structured data elements such as links to study registrations, data, and code. Structured data requirements help authors understand exactly what they need to provide, and structured data enables automatic checking during the submission process to promote uniform policy adherence. Structured data could also enable efficient and scalable monitoring of journals’ implementation of the TOP Statement [12].

Ultimately, policies and procedures aim to increase transparent and open practices in journal articles. For example, many journal policies state that all clinical trials must be registered prospectively to be considered for publication; while some authors will adhere to registration policies even in the absence of journal enforcement, other authors might submit unregistered trials, and unregistered trials could be published if journals do not have procedures to check and to enforce their policies. Thus, assessing the transparency of journal articles is the best way to assess the outcomes of journal policies and procedures. The TOP Statement provides a structured template for scientific publications to disclose the use of open science practices in a manner consistent with policies and procedures that aim to increase transparency and openness [12].

### Objectives

This manuscript describes processes and instruments for evaluating journal implementation of the TOP Guidelines. We developed this process as part of the Transparency of Research Underpinning Social Intervention Tiers (TRUST) Initiative to advance open science in the social intervention research ecosystem. To demonstrate the application of these processes and instruments, we will evaluate journals that have published social intervention research used by federal evidence clearinghouses, which is research intended to inform evidence-based social policy [13]. We will demonstrate how to calculate the “TOP Factor” for each eligible journal using a structured instrument. Then, we will demonstrate how to use structured instruments to assess each journal’s procedures and practices, and whether those procedures and practices are consistent with their stated policies. Throughout the study, we also will assess the interrater agreement (IRA) and the interrater reliability (IRR) of the structured instruments, and we will identify challenges to assessing and implementing standards in the TOP Guidelines. Ultimately, we aim to facilitate use of these processes and instruments in future studies and interventions, and to support clarifications and improvements to the TOP Guidelines.

## Methods

**Fig. 2:**
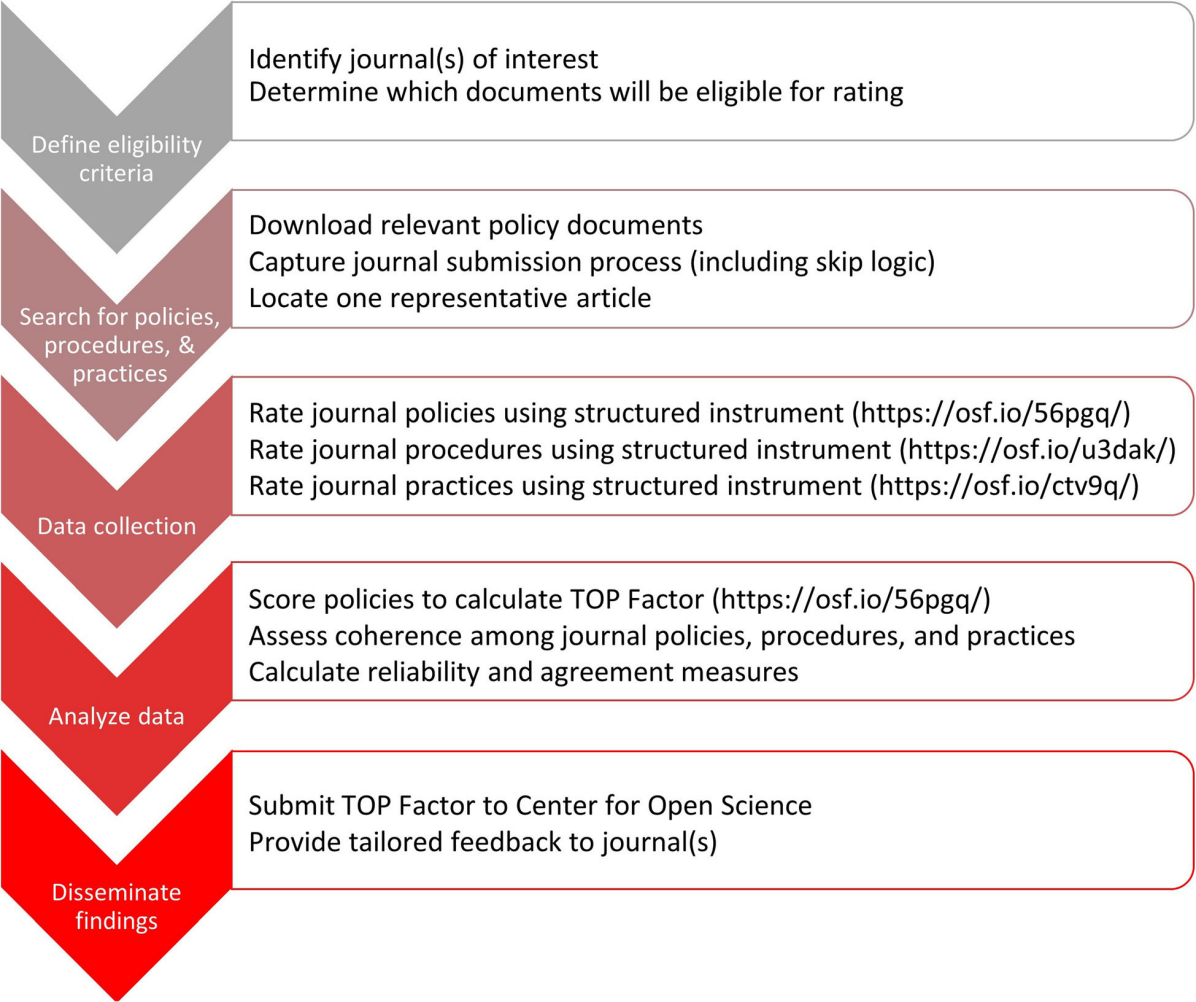
TRUST Process for rating journal policies, procedures, and practices

The overall TRUST Process for rating journal policies, procedures, and practices according to the TOP Guidelines is summarized in Fig. 2.

Although there are no reporting guidelines for a methodologic study of this kind, the TRUST Process follows best-practices for research synthesis that are applicable to our study. For example, we will identify and assess eligible policies, procedures, and practices using methods similar to article identification and data extraction in systematic reviews [14]. We will report this information about document identification, eligibility decisions, and results following the Preferred Reporting Items for Systematic Reviews and Meta-Analyses (PRISMA) guidelines [15]. To assess agreement and reliability of the rating instruments used in the TRUST Process, we will calculate the IRA and IRR for individual items in each instrument and for the overall level of implementation (0, 1, 2, or 3) of each of the ten standards in the TOP Factor. We will report psychometric information about the rating instruments following the Guidelines for Reporting Reliability and Agreement Studies (GRRAS) [16].

### Eligibility criteria for journals

Before identifying eligible journals, we searched for federal evidence clearinghouses in a previous study [13]. Clearinghouses rate the quality of published empirical studies on the effects of social interventions to distinguish and disseminate information about “evidence-based” interventions [13]. We identified 10 evidence clearinghouses funded by the United States federal government that review intervention research used for decision-making by the departments of Education, Health and Human Services, Justice, and Labor.

In the current study, we will include all journals that published at least one report of an evaluation used by one federal clearinghouses to support the highest rating possible for an intervention (i.e., a “top tier” evidence designation). We will include journals that have changed publisher or changed name since publishing an eligible report. We will exclude journals that have ceased operation entirely.

### Inclusion and exclusion criteria for policy documents (instructions to authors) for eligible journals

For each eligible journal, two trained graduate research assistants will independently search the journal website for its “Instructions to Authors” and other policy documents. We will identify policy documents that might describe recommendations and requirements related to research transparency and openness. Although open access publishing and preprints are related to transparency and openness, we will not address these issues because they are not addressed in the TOP Guidelines or TOP Factor.

To be eligible, policy documents must be publicly available on a journal website or listed on the journal website as available by request (e.g., from the publisher or editor). Our objective is to describe each journal’s current policies, which might differ from the policies in place when those journals published research used by eligible evidence clearinghouses. Because our objective is to rate these documents per se, we will not contact editors or publishers to clarify policies that are not described in publicly available documents.

For each eligible journal, the graduate research assistants will independently identify eligible documents. Each graduate research assistant will have a folder with their name on Google Drive; for each journal, they will download and save dated copies of websites and other policy documents found on the journal websites (i.e., as PDF files in a subfolder named using the journal’s title). Each pair of graduate research assistants will then meet and compare the identified documents. Disagreements about the eligibility of policy documents will be resolved through discussion. Any unresolved disagreements will be reconciled by consulting the principal investigators and by consulting additional team members during weekly progress meetings. Any additional documents and information regarding journal policies that are identified during the rating process will be downloaded and saved in a subfolder (i.e., indicating that they were found after the initial search). If policy documents cannot be obtained by searching online, we will contact the journal editors or administrators up to three times to request journal policy documents.

We will rate only policy documents that are specific to each journal; although we will note their existence, we will not rate linked policies on external websites. For example, we will consider specific language in journal policies that implements International Committee of Medical Journal Editors (ICMJE) recommendations, but we will not consider a journal policy to incorporate ICMJE recommendations merely by referencing or linking to the ICMJE website. Similarly, we will not rate society or publisher policies that are not specifically incorporated in a journal’s policies because such policies might not be applied equally by all journals affiliated with the society or publisher. For example, American Psychological Association (APA) journals might refer to the APA Publication Manual. We will consider a journal policy to include APA policies that are described specifically in the journal’s instructions to authors and other journal policy documents; however, we will not rate the APA Publication Manual for each APA journal because some APA journals might not incorporate all of its recommendations.

### Inclusion and exclusion criteria for procedures (manuscript submission systems) for eligible journals

For each eligible journal, we will identify procedures that promote transparency and openness. A trained graduate research assistant will initiate a manuscript submission through the journal’s electronic submission system. The graduate research assistant will create a journal account and simulate each submission step in the submission process using blank “dummy” files. They will take screenshots of each step, which they will download and save for assessment. They will download and save submission instructions for journals that do not have electronic submission systems (e.g., journals that require manuscript submission by email).

Because manuscript submission systems might ask questions related to transparency and openness depending on answers to previous questions (“display logic”), the graduate research assistant will answer questions such that all relevant questions and fields would appear. For example, if a manuscript submission system asks whether a study was registered, they will select “Yes” for the purpose of eliciting additional questions about the registration (e.g., the registration number). They will capture all dropdown menus and other options as screenshots. They also will proceed with submission steps without selecting items or filling in fields for the purpose of evoking alerts that would identify which fields are required.

All graduate research assistants tasked with identifying journal procedures will discuss issues related to the eligibility of procedures, and issues related to answering questions during the submission process, with each other and with the principal investigators during weekly progress meetings.

### Inclusion and exclusion criteria for practices (published articles) for eligible journals

We will search for articles published in eligible journals between January 1, 2020 and June 30, 2020 (inclusive). For journals in which no eligible articles on intervention research can be found between January 1, 2020 and June 30, 2020, we will search articles published between July 1, 2019 and December 31, 2019. For the purpose of this study, we will consider the date of publication to be the issue to which each article was assigned rather than other dates associated with articles such as the date of acceptance or the date of publication online ahead of print. For example, an article published online ahead of print in December 2019 and appearing in a January 2020 issue would be eligible, while an article published online ahead of print in June 2020 and appearing in the July 2020 issue would be ineligible.

Two trained, independent graduate research assistants will screen the titles and abstracts of potentially eligible articles, and they will enter citation information (i.e., volume number, issue number, first page number, and DOI) using a Research Electronic Data Capture (REDCap) form (See Additional file 1). All articles identified by either graduate research assistant will be retrieved for full-text review. A principal investigator will then review full-texts and identify one eligible article per journal (Additional file 2). Questions about inclusion will be resolved through discussion with the other principal investigator.

For our study of approximately 345 influential journals, we aim to include articles reporting “social intervention research.” We define “social intervention research” as studies evaluating the effectiveness of deliberate actions intended to modify processes and systems that are social and behavioral in nature (such as cognitions, emotions, norms, relationships, and environments) and are hypothesized to improve health or social outcomes [17]. If no social intervention research articles are found, we will look for an article describing other quantitative research to which TOP would be applicable (e.g., randomized and non-randomized studies designed to understand basic social or behavioral processes). We will exclude studies that are qualitative only, and we will exclude reports that do not include the results of evaluations (e.g., protocols, reports describing the baseline characteristics of participants in an evaluation, case studies, systematic reviews and meta-analyses). We will exclude studies that evaluate medical interventions, including studies that compare social and behavioral interventions with drugs (including nicotine replacement and electronic cigarettes), biologics, medical devices, nutritional supplements, and surgeries.

### Data collection: journal characteristics

We will collect descriptive information about each journal from Journal Citation Reports, 2019 [18], including: Web of Science Categories [19], Publisher, Rank in Category, Impact Factor, 5-year Impact Factor, and Article Influence Score. We will use the COS database [20] to identify whether a journal is recognized by COS as a TOP signatory.

### Data collection: instruments for rating policies, procedures, and practices

To develop each rating instrument, the principal investigators drafted a list of questions organized by standards in the TOP Guidelines [8, 21]. To promote their reproducibility and scalability, each instrument includes factual “Yes/No” questions and detailed instructions. We will use REDCap to rate journal policies and procedures, and EPPI-Reviewer to rate journal practices. To promote efficiency and to ensure consistency of the data, the instruments will use skip logic; all raters will rate a minimum set of items, and raters may rate additional items depending on their answers. Raters will not be masked to journal names.

The principal investigators sought feedback about each preliminary rating instrument from colleagues at the COS. Next, the principal investigators trained the graduate research assistants by introducing the project aims, answering general questions about transparency and openness, and discussing each item on the preliminary instruments. The principal investigators then selected a small number of journal policies, procedures, and published articles to be rated by multiple graduate research assistants for pilot testing.

During the pilot testing phase for each instrument, graduate research assistants rated a small number of items and then discussed the clarity of the items, instructions, and challenges encountered when rating. Ahead of each weekly project meeting, one project coordinator calculated the proportion of journals with disagreements for each item; the principal investigators reviewed that report and discussed reasons for disagreements and ways to improve the instrument (e.g., reduce ambiguity in the question wording, add examples, include instructions for boundary cases). For each instrument, we repeated the pilot testing process until we had addressed all outstanding questions and obtained satisfactory levels of agreement for all items.

We will use the rating instruments to assess policies, procedures, and practices. To assess journal policies, each journal will be assigned to three graduate research assistants to be rated using an online form (Additional file 3), which includes a link to instructions for completing the form. Skip logic and data codebook for the policy data are available as Additional file 4. The policy REDCap project XML file can be used to reproduce these processes (Additional file 5). The TOP Standards levels will be calculated using the journal policy TOP scoring document (Additional file 6).

Because we anticipate that journal procedures and published articles may be less complicated and more objective to rate compared with journal policies, two (rather than three) graduate research assistants will independently rate procedures and published articles using online forms. The rating form, skip logic and data codebook, and REDCap project XML file for journal procedures are available as Additional files 7, 8, 9. The rating form for journal practices is available as Additional file 10.

As with journal policies, graduate research assistants will assess journal procedures saved in folders on Google Drive. Instruments for assessing procedures and published articles will focus on the information in those sources only; for example, raters will assess whether articles report that studies were registered and whether data are available, but raters will not confirm each study’s registration status or confirm that they can reproduce the results in an article using publicly available data.

During the data collection phase, the team will continue to meet weekly to review and discuss disagreements and to address any new questions or problems that arise with the rating process. Disagreements between raters will be resolved by one of the principal investigators. PIs will review the items where there are discrepancies among the raters and reconcile these disagreements. For each policy, procedure, and practice, we will produce a record with the final ratings for analysis.

### Methods of analysis: scoring journal policies, procedures, and practices

**Fig. 3:**
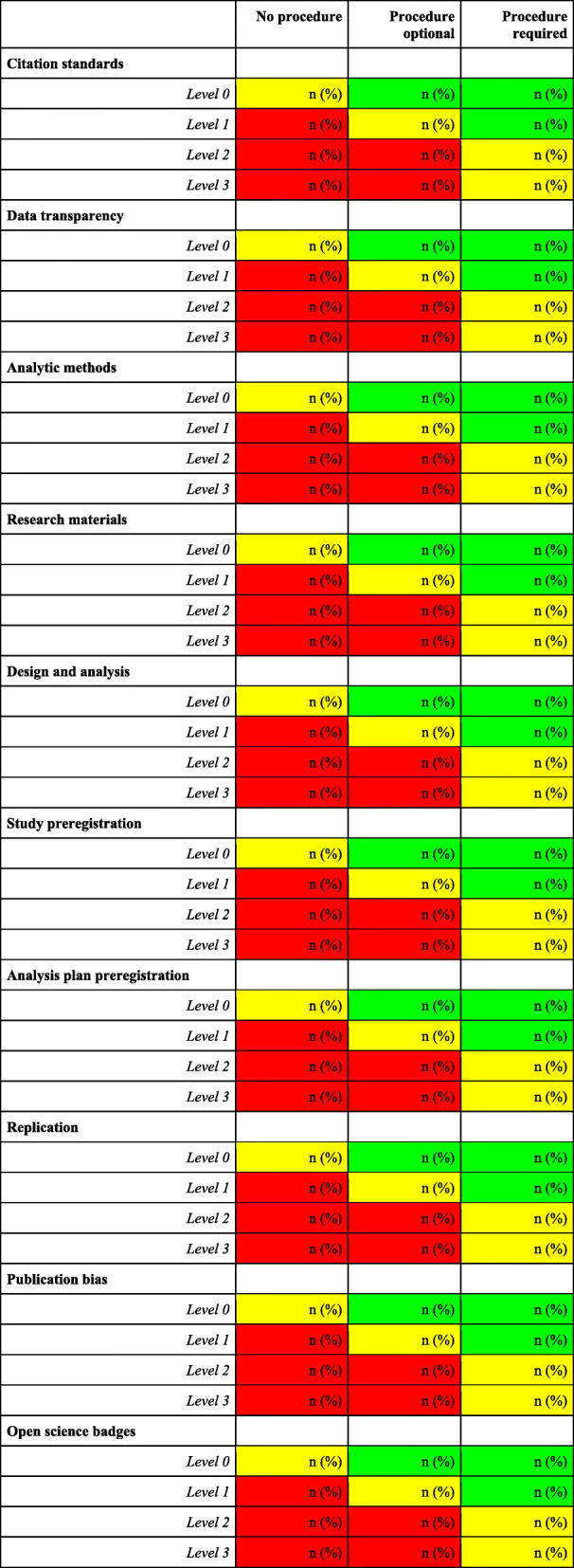
Template for figure describing concordance between policies and procedures for promoting transparency and openness. Green cells: Procedures include requirements for transparency and openness that exceed requirements stated in the journal policy. Yellow cells: Policies and procedures are concordant with respect to transparency and openness. Red cells: Policies describe transparency and openness requirements that are not supported by corresponding procedures

For each journal, we will calculate the TOP Factor based on the policy rating instrument using an algorithm to determine the level at which each journal follows each standard (i.e., Level 0 to Level 3). We will not assign “levels” to procedures and practices because TOP Factor is designed to evaluate journal policies and does not address journal procedures and practices directly (thus, there are no corresponding “levels” to assign). Instead, we will report whether the journal’s procedure for each standard: does not exist; exists but is not required; or exists and is required. We will then compare the degree to which there is alignment of the journal’s policies with their procedures and practices (see Fig. 3).

Based on previous meta-research concerning transparency and openness in social and behavioral intervention science [22, 23], we hypothesize that most journal policies will be rated Level 0 or Level 1 for most of the ten TOP Factor standards. We also expect to find differences across standards (for example, journals might be more likely to require study registration compared with data sharing). We expect that few submission systems will promote implementation of the TOP Guidelines, and we expect that many journals’ procedures will not facilitate their stated policies. Finally, we hypothesize that most journal practices will fail to adhere to normative standards for transparency and openness, and we expect that journal practices will not align consistently with journal policies.

### Methods of analysis: reliability of the rating instruments

For each rating instrument, we will assess IRA and IRR for the items that must be rated by all graduate research assistants (i.e., items that are displayed for all raters and for all journals); we will not assess IRA and IRR for items that can be skipped. Reliability is a correlational and proportional consistency measure used to determine whether ratings vary between individuals who assess the same thing (i.e., policy, procedure, practice) and how much of this variability is due to error [24, 25]. Agreement indicates the interchangeability among raters, “the extent to which raters make essentially the same ratings” [25]. Although reliability and agreement may be related, it is possible to have high reliability and a low agreement or low reliability and a high agreement, though the latter occurs rarely [26]. Thus, we will report both measures and interpret them together.

We will assess the IRA using the proportion of overall agreement (i.e., proportion of cases for which all raters agree) and the proportion of specific agreement (i.e., observed agreement relative to each of the “Yes” and “No” rating categories) [27, 28]. The level of measurement for each of the items in the policy, procedure, or practices instrument is on a nominal scale (“Yes” or “No”), and different groups of investigators will rate different journals. Hence, we will use Fleiss’ kappa statistic [29] to evaluate the IRR for each item. We will also report the 95% confidence intervals for IRR and IRA measures.

Because each of the standards in the TOP Guidelines is rated using an ordinal scale, we will use the intraclass correlation coefficient (ICC) to evaluate the IRR for each of the standards [16, 30]. ICC can be estimated using different models [24, 31]; we will use the two-way random effects model in which we treat both the journals and the investigators as random effects [30, 31], we will use the “single rater” type and “absolute agreement” definition [24]. To evaluate the IRA for standards, we will calculate the proportions of overall and specific agreement [16]. We will report the 95% confidence interval for agreement and ICC.

The magnitude of the kappa statistic shows “the proportion of agreement greater than that expected by chance” [32]. The magnitude can range from − 1 to 1, with values below 0 representing poor agreement, 0 representing agreement that is not better than that expected by chance, and 1 representing perfect agreement [33] (Table 2). This magnitude is influenced by the prevalence of an attribute and by the extent to which raters disagree on the prevalence of that attribute [32]. For example, a low prevalence of “Yes” responses for an item would result in a high proportion of agreement but reduced kappa. Furthermore, disagreement between investigators on the proportion of “Yes” and “No” responses will increase kappa [32]. Nonindependent ratings can also inflate kappa; in this study, the investigators will discuss questions during pilot testing and throughout the study, but investigators will rate items independently and will not discuss specific ratings until completed. Lastly, to facilitate the interpretation of Fleiss’ kappa statistics, we will report the prevalence of each attribute along with kappa for each item.

**Table 2:** Interpretation of strength of agreement for intraclass correlation coefficient (ICC) and kappa statistics adapted from [24, 33]

Value	Kappa	ICC
≤0.00	Poor	Poor
0.01–0.10	Slight	Poor
0.11–0.20	Slight	Poor
0.21–0.30	Fair	Poor
0.31–0.40	Fair	Poor
0.41–0.50	Moderate	Poor
0.51–0.60	Moderate	Moderate
0.61–0.70	Substantial	Moderate
0.71–0.80	Substantial	Good
0.81–0.90	Almost perfect	Good
0.91–1.00	Almost perfect	Excellent

We will follow interpretation guidelines developed for ICC (the IRR measure for continuous data) (Table 2) [24]. Here, a low ICC could be due to a low degree of measurement agreement or low variability among the sampled journals. Because building confidence intervals improves interpretation [24], we will report the 95% confidence intervals for ICC measures.

#### Differences between raters

For each instrument (policy, procedure, practices), we will calculate the number of items that each graduate research assistant reviewed. For each graduate research assistant, we will calculate the proportion of items on which they agree with the final reconciled ratings as well as the sensitivity and specificity of each of their ratings (i.e., compared with the final reconciled ratings). Lastly, for the policy instrument, which will be rated by three graduate research assistants, we will also calculate the proportion of items for which each assistant’s rating is in the minority (i.e., their rating is different from the other two ratings).

### Methods of analysis: concordance of journal policies with procedures and practices

Because the TOP Guidelines were designed to improve journal policies, and the TOP Factor summarizes the transparency and openness of policies, levels in TOP apply to policies specifically. Consequently, we will present descriptive statistics concerning the concordance of policies with journal procedures (see Fig. 3). We also will review one article per journal and report the extent to which these articles disclose the use of each open science practice in TOP. Some characteristics of journal procedures and practices could be described ordinally, though there are no consensus-based “levels” for procedures and practices.

Data management will be done in Python (version 3.7.6, Python Software Foundation, Beaverton, OR, US) [34] and data analysis will be performed in R [35] using RStudio [36]. We will use the ‘obs.agree’ [37] and ‘irr’ [38] packages to estimate the IRA and IRR measures, respectively.

## Discussion

The TRUST Process provides systematic methods and rating instruments for assessing and scaling-up the appropriate implementation of the TOP Guidelines by journals to which each of the standards in TOP are applicable. By examining a large cohort of influential journals using this process, we will provide a comprehensive account of whether their policies, procedures, and practices are consistent with standards for open science and thereby facilitate the publication of trustworthy findings to inform evidence-based policy.

In addition to providing a method for evaluating implementation of the TOP guidelines, we expect that by using this process we will identify ways to refine the TOP Guidelines. Potential refinement could include improving templates for adoption in journal instructions to authors, manuscript submission systems, and article templates; revising explanatory guidance intended to enhance the use, understanding, and dissemination of the TOP Guidelines; and clarifying the distinctions among different levels of implementation.

## Supplementary Information

**Additional file 1.**

**Additional file 2.**

**Additional file 3.**

**Additional file 4.**

**Additional file 5.**

**Additional file 6.**

**Additional file 7.**

**Additional file 8.**

**Additional file 9.**

**Additional file 10.**

## Data Availability

Research materials are available on the Open Science Framework (https://osf.io/txyr3). Once completed, we will also share data and code on the Open Science Framework.

## Abbreviations

TOP: Transparency and Openness Promotion
COS: Center for Open Science
TRUST: Transparency of Research Underpinning Social Intervention Tiers
IRA: Interrater agreement
IRR: Interrater reliability
PRISMA: Preferred Reporting Ttems for Systematic Reviews and Meta-Analyses
GRRAS: Guidelines for Reporting Reliability and Agreement Studies
ICMJE: International Committee of Medical Journal Editors
APA: American Psychological Association
REDCap: Research Electronic Data Capture
ICC: Intraclass correlation coefficient
