# Supplementary material for: Evaluating implementation of the Transparency and Openness Promotion (TOP) guidelines: the TRUST process for rating journal policies, procedures, and practices
Source: Res Integr Peer Rev. 2021 Jun 2;6:9. doi: 10.1186/s41073-021-00112-8 (PMC8173977; doi:10.1186/s41073-021-00112-8)
Supplement: Supplementary file 1 — Additional file 1. [file 41073_2021_112_MOESM1_ESM.pdf]

Additional file 1: Article Screening Form

Complete this form following the instructions here [Instructions are included at the end of this Additional file].

References are normally formatted as “Volume(Issue): Page”. For the reference here, which includes “20(1): 553”, the volume is “20” the issue is “1” and the first page is “553”. For electronic journals, such as this reference to “8(5):e65442”, the volume and issue are the same (i.e., volume “8” and issues “5”) and the page number takes a slightly different format, “e65442”.

Enter the DOI name. For the reference above, you would enter “10.1186/s13063-019-3581-3” (note that this is not a link, and there is no period at the end).

If an artilce does not have a DOI, please include the article's web address in the DOI field (the whole address including "https://"). Below are some resources for locating an article's DOI name:

- Google search: Enquote the article's title and paste it into Google search bar; Usually one of the top results has the DOI name.

- Onesearch

- PubMed

Please choose your name from the drop-down menu.

* must provide value

JOURNAL ASSIGNMENTS:
Please select one of the journals from the driop-down menu.
Please use "Journal_Article_Extraction_Assignments" on Google Drive to see your assignments.

* must provide value

You are reviewing _____

Please fill in the following fields;

Volume number

* must provide value

Issue number

Please do not include the parentheses

First page number

* must provide value

DOI

* must provide value

Please do not include "https://doi.org/"

If you have completed your article identification for _____, please remember to update the "Journal_Article_Extraction_Assignments" by changing the status column to the right of your name from "Assigned" to "Complete."

Journal_Article_Extraction_Assignments

Submit

Save & Return Later

Article Identification Form
Drafted April 23, 2020, Revised July 20, 2020

Criteria for journal issues Review all issues of assigned journals from January 1, 2020 through and including June 30, 2020. Include articles based on the date of the journal issue publication (not the first date of the electronic publication).

Criteria for journal articles Include only articles in paginated issues (i.e., articles that have been assigned an issue number and page number, which might include an electronic page number for online-only journals). Exclude articles that are available only "ahead of print" or "online early", and that have not been assigned to an eligible issue. Include all article types (e.g., "brief reports") should be included.

Criteria for studies Complete the article identification form for all eligible articles. Articles are eligible if they report an evaluation of the efficacy or effectiveness of a social or behavioral intervention, as defined below:

"Evaluations" (or "studies") use newly collected data (e.g., trials done by the authors) or existing data (e.g., publicly available datasets) but exclude reviews (e.g., narrative review, meta- analysis).

"Efficacy or effectiveness" means that evaluations must be quantitative (not only qualitative) and report outcomes (not a protocol, not only baseline characteristics or a description of the process for evaluating or delivering the intervention). Case studies (e.g., results for one person) should be excluded.

"Social or behavioral interventions" are activities that target social or behavioral processes, such as increasing participant motivation, to prevent or improve health or social outcomes. The term "social or behavioral" is meant to indicate that we will *exclude* evaluations that assign people to pharmaceuticals or medical devices in one or more groups.

- Eligible interventions include programs, practices, and products intentionally designed to lead to positive changes in outcomes of interest.
- Eligible processes (intervention mechanisms of action) include cognitions, emotions, behaviours, norms, relationships, and environments
- Eligible health outcomes include but are not limited to measures of physical, mental, and behavioral health or well-being.

- Eligible social outcomes include but are not limited to measures of crime, economics, education, employment, family and child welfare, social well-being, and substance use.
- Examples of social and behavioral interventions can be found in the following documents:
    - https://static-content.springer.com/esm/art%3A10.1186%2Fs13063-018-2735-z/MediaObjects/13063_2018_2735_MOESM2_ESM.docx
    - https://static-content.springer.com/esm/art%3A10.1186%2Fs13063-018-2735-z/MediaObjects/13063_2018_2735_MOESM3_ESM.docx

The term “interventions” is meant to be understood broadly; however, we will exclude studies of experimental manipulations that are designed to improve our understanding of basic behavioral or psychological processes rather than improve outcomes for individuals or populations. We will exclude studies of exposure (e.g., parental incarceration) that are not done to improve health and/or social outcomes.
