# Supplementary material for: Evaluating implementation of the Transparency and Openness Promotion (TOP) guidelines: the TRUST process for rating journal policies, procedures, and practices
Source: Res Integr Peer Rev. 2021 Jun 2;6:9. doi: 10.1186/s41073-021-00112-8 (PMC8173977; doi:10.1186/s41073-021-00112-8)
Supplement: Supplementary file 2 — Additional file 2. [file 41073_2021_112_MOESM2_ESM.pdf]

Additional file 2: Article Eligibility Form (Full Text)

1. Does the reported study evaluate an “intervention”?
   - An “intervention” is a deliberate set of actions intended to improve outcomes of interest for individuals or populations.
     - Exclude studies of experimental manipulations that are not designed to improve outcomes described below (e.g., studies in which participants complete a task and outcomes are assessed in a laboratory setting over a matter of hours). Experimental studies are not eligible if they are designed to improve our understanding of basic biological, psychological, or social processes (rather than improve outcomes for individuals or populations).
     - Exclude observational studies of exposure (e.g., studies about the association between participant characteristics or behaviors and outcomes). Intervention evaluations can use newly collected data (e.g., trials done by the authors) or existing data (e.g., publicly available datasets).
     - Exclude review articles (e.g., systematic reviews, narrative reviews), even if they report empirical data (e.g., meta-analysis).
2. Does the study evaluate the “efficacy or effectiveness” of the intervention? ○ “Efficacy” and “effectiveness” indicates that the study evaluated the effects of the intervention on outcomes of interest using quantitative data.
     - Exclude studies that report one or more of the following without also reporting quantitative outcome data: a protocol, baseline characteristics, a description of the process for delivering the intervention, or qualitative data on outcomes.
     - Case studies (e.g., results for one person) should be excluded.
     - Exclude “feasibility” studies designed to determine whether or how to conduct a larger trial to evaluate the effectiveness of an intervention.
3. Is the evaluated intervention “social or behavioral”?
   - “Social and behavioral interventions” are purposeful actions intended to improve outcomes of interest by modifying processes and systems that are social and behavioral in nature (e.g., knowledge, perceptions, attitudes, emotions, norms, relationships, and environments).
     - Exclude studies that report on interventions that assign human participants to groups receiving a pharmacological intervention, medical device, or physiotherapy.
   - Social and behavioral interventions might focus on: criminological, economic, educational, financial, health, and social outcomes.
