# Supplementary material for: Evaluating implementation of the Transparency and Openness Promotion (TOP) guidelines: the TRUST process for rating journal policies, procedures, and practices
Source: Res Integr Peer Rev. 2021 Jun 2;6:9. doi: 10.1186/s41073-021-00112-8 (PMC8173977; doi:10.1186/s41073-021-00112-8)
Supplement: Supplementary file 3 — Additional file 3. [file 41073_2021_112_MOESM3_ESM.pdf]

Additional file 3: Policy data collection form

Please choose your name from the drop-down menu.	◯ 1. Rater 1 name<br>◯ 2. Rater 2 name<br>◯ 3. Rater 3 name
[username], please type your first name in the box.	
JOURNAL ASSIGNMENTS:<br>Select one of the journal assignments. Please use the link below to find the Google spreadsheet titled "Journal Policy Data Extraction Assignments" that includes your journal assignments.<br><br>Journal Policy Data Extraction Assignments<br><br>Note: Once you have completed your assigned journals, please update the status column to the right of your name from "Assigned" to "Complete." Contact [redacted] for further instructions.	◯ Journal names

You are reviewing [jn]

CITATION STANDARDS (PREVIOUSLY COLLECTED DATA)

1a. For previously collected datasets described in a manuscript, does the policy describe a format for citing the DATASETS in the text and for listing them in the reference section (e.g., specific information to include)?

Do not consider data sharing as part of a new study. Journals might refer to this as “data citation” or “citation of existing data”. Answer “Yes” if the policy explains how to cite a dataset and/or includes examples of appropriate citations; for example, instructions might indicate that manuscripts should cite datasets in the text and list specific information in the reference section, such as name of the investigator, date of access, and URL. Answer “No” if the policy refers only to including a DOI or URL in the text. Answer “No” if the policy references publisher guidelines or a standalone style manual that includes examples of citations to datasets (e.g., AMA, APA) but the journal policy documents (i.e., instructions to authors and other documents on the journal website) neither include a format for citing previously collected datasets nor require dataset citation using a specific format (e.g., the policy does not require that datasets, specifically, be cited and that datasets be cited using AMA formatting). Answer “No” if the policy requires or encourages data citation but does not provide a citation format. Answer “No” if the policy addresses overlap with previous publications but does not describe how to cite data used for a study.

◯ Yes
◯ No

1b. Does the policy describe a format for citing CODE in the text and for listing it in the reference section (e.g., specific information to include)?

For this question, do not consider sharing code written as part of a new study. Code might be called a “program”, but “program” might also refer to software; you should answer “No” if the policy refers only to referencing software packages and commands (e.g., Stata, package “netmeta Version 1.1-0” in R). Answer “Yes” if the policy explains how to cite code and/or includes examples of appropriate citations; for example, instructions might indicate that manuscripts should cite databases in the text and list specific information in the reference section, such as name of the investigator, date of access, and URL. Answer “No” if the policy references publisher guidelines or a standalone style manual that includes examples of citations to code (e.g., AMA, APA) but the journal policy documents (i.e., instructions to authors and other documents on the journal website) neither include a format for citing code nor require code citation using a specific format (e.g., the policy does not require that code, specifically, be cited and that code be cited using AMA formatting). Answer “No” if the policy describes a format for citing and referencing data but does not explicitly mention citing/referencing code.

◯ Yes
◯ No

1c. Does the policy indicate that DATA CITATIONS should direct users to a dataset (i.e., citations refer to the location of a database)?<br><br>Answer "Yes" if the policy requires that the citation include the URL, DOI, or other persistent identifier. Answer "No" if the policy would allow citations to descriptions of databases rather than their locations.	◯ Yes<br>◯ No
1d. Does the policy require as a condition of publication that DATA CITATIONS (i.e., for data used in each manuscript) appear in the list of references?<br><br>For this question, consider only data that are being re-used in a study; do not consider data gathered as part of a new study. Answer "No" if the policy allows URLs or other descriptions in the text of manuscripts but does not require that citations appear in the list of references.	◯ Yes<br>◯ No
1e. Does the policy state that the journal will verify whether citations to DATASETS conform to these standards as a condition of publication?	◯ Yes<br>◯ No
1f. Does the policy indicate that code citations should direct users to the CODE (i.e., citations refer to the location of the code)?<br><br>Answer "No" if the policy would allow citations to descriptions of code rather than its location.	◯ Yes<br>◯ No
1g. Does the policy require as a condition of publication that CODE CITATIONS (i.e., for code used in each manuscript) appear in the list of references?<br><br>For this question, consider only data that are being re-used in a study; do not consider data gathered as part of a new study. Answer "No" if the policy allows URLs or other descriptions in the text of the manuscript but does not require citations in the list of references.	◯ Yes<br>◯ No
1h. Does the policy state that the journal will verify whether citations to CODE conform to these standards as a condition of publication?	◯ Yes<br>◯ No
1i. Directly copy and paste information about citation standards for DATASETS and CODE from the policy documents.<br><br>Copy entire sections that are relevant. Copy verbatim text and use quotation marks; do not otherwise edit the text (e.g., there is no need to remove line breaks or to edit characters that do not copy correctly). For multiple quotations, separate each using the word AND (capitalized).	(Citation Standards text)

DATA TRANSPARENCY (NEWLY COLLECTED DATA)

2a. For newly collected data (i.e., collected as part of a study submitted to this journal), does the policy require as a condition of publication that authors post the data on a repository for one or more types of studies?

Consider data gathered during a study. Although you should not consider data that are being re-used, you should assume that policies referring to "data" mean "newly collected data" unless otherwise specified. Repositories are permanent collections such as FigShare, Dryad, and institutional repositories. Answer "Yes" if the policy uses the word "require" or a synonym indicating that the data must be posted on a repository. Answer "No" if the policy "suggests", "strongly encourages", or otherwise mentions but does not mandate posting data. Answer "No" if the policy requires sharing data but does not specify that the data must be shared in a permanent collection.

◯ Yes
◯ No

2b. For newly collected data (i.e., collected as part of a study submitted to this journal), does the policy require that manuscripts indicate whether data are publicly available?

Answer "Yes" if the policy requires that authors indicate in their manuscripts whether data will be made available to other researchers. This might be called a "data sharing" "data availability" or "data access" statement. Answer "Yes" if the policy applies to one or more types of studies. Note that a "statement" would indicate whether or not data are available; a data sharing statement could indicate that data are not available. Answer "No" if the policy encourages but does not require this information. Answer "No" if the policy says nothing.

◯ Yes
◯ No

2c. Does the data transparency policy apply to all studies?

Answer "No" if the policy applies to only certain types of studies e.g., clinical trials.

◯ Yes
◯ No

2d. Directly copy and paste information about DATA TRANSPARENCY (NEWLY COLLECTED DATA) from the policy documents.

Copy entire sections that are relevant. Copy verbatim text and use quotation marks; do not otherwise edit the text (e.g., there is no need to remove line breaks or to edit characters that do not copy correctly). For multiple quotations, separate each using the word AND (capitalized).

ANALYTIC METHODS (CODE) TRANSPARENCY

3a. Does the policy require as a condition of publication that CODE used to perform statistical analyses reported in each manuscript be posted on a repository for one or more types of studies?

Repositories are permanent collections such as FigShare, Dryad, and institutional repositories. Code might be called a “program”, but “program” might also refer to software; you should answer “No” if the policy refers only to referencing software packages and commands (e.g., Stata, package “netmeta Version 1.1-0” in R). Answer “Yes” if the policy uses the word “require” or a synonym indicating that the code must be posted on a repository. Answer “No” if the policy “suggests”, “strongly encourages”, or otherwise mentions but does not mandate posting code. Answer “No” if the policy requires code sharing but does not specify that code must be shared in a permanent collection.

◯ Yes
◯ No

3b. Does the policy require that each manuscript indicate whether the code used to produce the results in that manuscript is publicly available?

Answer “No” if the policy encourages but does not require this information. Answer “No” if the policy says nothing.

◯ Yes
◯ No

3c. Does the analytic methods (code) transparency policy apply to all studies?

Answer “No” if the policy applies to only certain types of studies e.g., clinical trials.

◯ Yes
◯ No

3d. Directly copy and paste information about ANALYTIC METHODS (CODE) TRANSPARENCY from the policy documents.

Copy entire sections that are relevant. Copy verbatim text and use quotation marks; do not otherwise edit the text (e.g., there is no need to remove line breaks or to edit characters that do not copy correctly). For multiple quotations, separate each using the word AND (capitalized).

VERIFICATION OF DATA AND ANALYTIC METHODS

4a. Does the policy state that analyses will be reproduced independently prior to publication for one or more types of studies?

◯ Yes
◯ No

4b. Does the verification policy apply to all studies?

Answer “No” if the policy applies to only certain types of studies e.g., clinical trials.

◯ Yes
◯ No

4c. Directly copy and paste information about the VERIFICATION OF DATA AND ANALYTIC METHODS from the policy documents.

Copy entire sections that are relevant. Copy verbatim text and use quotation marks; do not otherwise edit the text (e.g., there is no need to remove line breaks or to edit characters that do not copy correctly). For multiple quotations, separate each using the word AND (capitalized).

RESEARCH MATERIALS TRANSPARENCY

5a. Does the policy require that each manuscript disclose whether newly created research materials are available to the public for one or more types of studies?

Research materials refer to specific items that would be necessary for others to conduct an independent replication of the research, such as those used in the intervention (e.g. instructional materials, proprietary computer programs, etc.) or for data collection (e.g. questionnaires, interview guides). For this question, consider only materials created for the current study; do not consider commercially available tools (e.g., statistical analysis programs) or materials that are being re-used from previous studies. Answer “Yes” if the policy uses the word “require” or a synonym indicating that the availability of materials must be disclosed. Answer “No” if the policy “suggests”, “strongly encourages”, or otherwise mentions but does not mandate that manuscripts indicate whether research materials are available.

○ Yes
○ No

5b. For physical materials, does the policy require that each manuscript include persistent identifiers?

For this question, consider physical objects such as drugs, biospecimens, or physical apparatuses; do not consider materials that could be shared digitally such as apps or websites. Persistent identifiers are defined here: https://www.rd-alliance.org/sites/default/files/PID-re port v6.1 2017-12-13 final 0.pdf

○ Yes
○ No

5c. For materials that can be shared digitally, does the policy require that authors share newly created research materials?<br><br>For this question, consider only materials created for the current study; do not consider materials that are being re-used for a new study. Do not consider physical materials. Instead, consider only materials that can be shared digitally, such as: images, diagrams, questionnaires, computer programs, and websites. Answer “Yes” if the policy includes a requirement but allows exceptions for legal, ethical, or physical reasons (e.g., ethical concerns about identifying patients in medical research, inability to share many terabytes of data on existing repositories). Answer “No” if the policy allows non-disclosure because of commercial interests or other interests unrelated to applicable laws, ethical standards, or physical barriers.	◯ Yes<br>◯ No
5d. For materials that can be shared digitally, does the policy require that authors share newly created research materials in a permanent repository?<br><br>Repositories are permanent collections such as FigShare, Dryad, and institutional repositories. Answer “No” if the policy requires materials sharing but does not specify that materials must be shared in a permanent collection.	◯ Yes<br>◯ No
5e. Does the policy state that the journal will verify whether citations to research materials are working and that the materials are accessible?	◯ Yes<br>◯ No
5f. Does the research materials transparency policy apply to all studies?<br><br>Answer “No” if the policy applies to only certain types of studies e.g., clinical trials.	◯ Yes<br>◯ No
5g. Directly copy and paste information about RESEARCH MATERIALS TRANSPARENCY from the policy documents.<br><br>Copy entire sections that are relevant. Copy verbatim text and use quotation marks; do not otherwise edit the text (e.g., there is no need to remove line breaks or to edit characters that do not copy correctly). For multiple quotations, separate each using the word AND (capitalized).	

DESIGN AND ANALYSIS TRANSPARENCY

6a. Does the policy reference or refer to one or more reporting guidelines (e.g., by reference to the EQUATOR Network, CONSORT, or a checklist of specific items used by the journal)?

Reporting guidelines describe the minimum information about study methods and results that should be included in a journal article. Reporting guidelines differ from “style guides” that describe how information should be reported (rather than what information to report). Most reporting guidelines include a checklist and a flow diagram and have an acronym or name (e.g., “JARS”). There may not be an appropriate reporting guideline for some studies. Answer “Yes” if the policy refers to reporting guidelines for one or more types of studies, or if the journal policy suggests that authors use a reporting guideline “if applicable”. Answer “No” if the policy refers to the TOP Guidelines, or to other guidelines related to research transparency, but the policy does not refer to guidelines for reporting research methods and results. Answer “No” if the policy says that reports must include complete information about their methods and results but the policy does not refer to a specific guideline for reporting studies.

◯ Yes
◯ No

6b. Does the policy require that one or more types of manuscript be accompanied by a completed checklist to demonstrate that the manuscript adheres to an applicable reporting guideline?

Answer “Yes” if the policy uses the word “require” or a synonym indicating that authors must submit a completed checklist with a quotation or page number corresponding to each item in the reporting guideline. Answer “No” if the policy says that authors must follow reporting guidelines but does not say that authors must submit a checklist. Answer “No” if the policy requires submission of a “flow diagram” but does not require submission of a completed checklist. Answer “No” if the policy “suggests”, “strongly encourages”, or otherwise mentions but does not mandate that one or more types of manuscript be accompanied by a completed checklist.

◯ Yes
◯ No

6c. Does the policy state that publication is conditional on the journal verifying adherence to reporting guidelines?

Answer “Yes” if the journal indicates that the editors or reviewers will check the manuscript for adherence and that only articles that adhere to reporting guidelines are eligible for publication.

◯ Yes
◯ No

6d. Does the reporting guidelines policy apply to all studies?

Answer “No” if the policy applies to only certain types of studies e.g., clinical trials.

◯ Yes
◯ No

6e. Directly copy and paste information about DESIGN AND ANALYSIS TRANSPARENCY from the policy documents.

Copy entire sections that are relevant. Copy verbatim text and use quotation marks; do not otherwise edit the text (e.g., there is no need to remove line breaks or to edit characters that do not copy correctly). For multiple quotations, separate each using the word AND (capitalized).

REGISTRATION OF STUDIES

7a. Does the policy require that one or more types of manuscript indicate whether the study was registered?

○ Yes
○ No

Answer "Yes" if the policy uses the word "require" or a synonym indicating that one or more types of studies must be registered, even if the policy does not specify that registration must be done prospectively. Answer "Yes" if the policy indicates that manuscripts must say whether or not the studies they describe were registered.

7b. Does the policy state that one or more types of manuscript must describe how to access the study registration (e.g., provide a registration number or URL), if the study was registered?

○ Yes
○ No

7c. Does the policy require prospective study registration as a condition of publication for one or more types of manuscript?

○ Yes
○ No

"Prospective" registration occurs before a key event, such as before enrolling the first participant, before completing data collection, or before analyzing the data. Answer "Yes" if the policy requires "prospective registration" or "preregistration." Answer "Yes" if the policy explains that one or more types of studies must be registered before beginning or completing enrollment, or before completing data collection.

7d. Does the policy state that one or more types of manuscript must be registered on a specific register (e.g., ClinicalTrials.gov, AEA RCT Registry, or a register endorsed by the World Health Organization (WHO))?

○ Yes
○ No

7e. Does the policy state that study registrations will be reviewed (e.g., by journal staff or editors) prior to publication to verify that the registrations adhere to minimum standards for describing planned methods and/or outcomes?

○ Yes
○ No

Answer "Yes" if the policy indicates that the editors or reviewers will check whether studies were actually registered and that the editors or reviewers will confirm whether one or more minimum requirements were met (e.g., registrations include minimum information about interventions or sample size).

7f. Does the study registration policy apply to all studies?

Answer “No” if the policy applies to only certain types of studies e.g., clinical trials.

◯ Yes
◯ No

7g. Directly copy and paste information about REGISTRATION OF STUDIES from the policy documents.

Copy entire sections that are relevant. Copy verbatim text and use quotation marks; do not otherwise edit the text (e.g., there is no need to remove line breaks or to edit characters that do not copy correctly). For multiple quotations, separate each using the word AND (capitalized).

REGISTRATION OF ANALYSIS PLANS

8a. Does the policy require that one or more types of manuscript indicate whether the analysis plan was registered?

Answer “Yes” if the policy requires analysis plan registration for at least one study type, even if the policy does not specify that registration must be done prospectively. Answer “Yes” if the policy indicates that manuscripts must say whether or not analysis plans were registered or if the policy requires that authors register analysis plans on a specific registry, provide a registration number, provide a DOI, or provide a URL. Answer “No” if the policy requires that authors submit the analysis plan (i.e., to the journal as part of the manuscript submission process) but the policy does not require that the analysis plan be registered on a separate register such as Open Science Framework or ClinicalTrials.gov.

◯ Yes
◯ No

8b. Does the policy state that one or more types of manuscript must describe how to access the registered analysis plan (e.g., provide a URL), if it was registered?

◯ Yes
◯ No

8c. Does the policy state that analysis plans will be reviewed prior to publication to verify that the registrations adhere to minimum standards for describing planned analyses?

Answer “Yes” if the policy requires that authors register analysis plans on a specific registry that conducts quality control. If the policy does not refer to a specific registry, answer “Yes” if the policy indicates that the editors or reviewers will check whether each analysis plan was actually registered and that the editors or reviewers will confirm whether one or more minimum requirements were met (e.g., the analysis plans contain specific types of information about the planned statistical methods).

◯ Yes
◯ No

8d. Does the policy require prospective registration of the analysis plan as a condition of publication for one or more types of manuscript?

"Prospective" registration occurs before a key event, such as before enrolling the first participant, before completing data collection, or before analyzing the data. Answer "Yes" if the policy requires "prospective registration" or "preregistration" of analysis plans, and answer "Yes" if the policy explains that analysis plans for one or more types of studies must be registered before beginning or completing enrollment, or before completing data collection.

◯ Yes
◯ No

8e. Does the analysis plan registration policy apply to all studies?

Answer "No" if the policy applies to only certain types of studies e.g., clinical trials.

◯ Yes
◯ No

8f. Directly copy and paste information about REGISTRATION OF ANALYSIS PLANS from the policy documents.

Copy entire sections that are relevant. Copy verbatim text and use quotation marks; do not otherwise edit the text (e.g., there is no need to remove line breaks or to edit characters that do not copy correctly). For multiple quotations, separate each using the word AND (capitalized).

REPLICATION, REGISTERED REPORTS & PUBLICATION BIAS

9a. Does the policy state that authors may submit manuscripts as "Registered Reports" to be peer reviewed prior to conducting the research and obtaining the results?

Answer "Yes" if the policy uses the term "Registered Report" or states that the journal will conduct a first stage of review prior to the data being collected (i.e., before the outcomes are observed) and make a preliminary decision about acceptance. Answer "Yes" if the journal allows "Registered Reports" for one or more types of study. Answer "No" if the policy makes no mention of "Registered Reports" or other ways to submit manuscripts for review before the results are obtained.

◯ Yes
◯ No

9b. What type of studies may be submitted as "Registered Reports"?

◯ Both original and replication studies
◯ Original studies only
◯ Replication studies only
◯ Unclear (not stated

9c. Does the policy state that authors may submit manuscripts to be reviewed based on the background and methods sections alone?<br><br>Answer “Yes” if the policy states authors can submit manuscripts that describe why and how studies were conducted, without results and discussion sections, as the first stage of review for any type of submission. This may be referred to as a “results-masked” or “results-blind” review. Answer “No” if the policy makes no mention of a results-blind review stage.	◯ Yes<br>◯ No
9d: What types of studies may be reviewed based on the background and methods alone?	◯ Both original and replication studies<br>◯ Original studies only<br>◯ Replication studies only<br>◯ Unclear (not stated
9e. Does the policy “welcome”,“encourage”, or otherwise indicate that the journal accepts replication studies?<br><br>“Replication” studies aim to reproduce the methods and results of previous studies, and are typically described using the term “replication”. Answer “Yes” if the policy refers to replications of specific types of studies (e.g., experiments) or article types (e.g., Registered Reports”).	◯ Yes<br>◯ No
9f. Does the policy state that the statistical “significance” of results or “novelty” of findings are not a criteria for publication decisions?<br><br>Answer “Yes” if the policy states that the nature of results (null, negative, positive) will be ignored or will not be factored into decisions about publishing manuscripts on original primary studies.	◯ Yes<br>◯ No
9g. Directly copy and paste information about REPLICATION, REGISTERED REPORTS, and RESULTS BLIND REVIEW from the policy documents.<br><br>Copy entire sections that are relevant. Copy verbatim text and use quotation marks; do not otherwise edit the text (e.g., there is no need to remove line breaks or to edit characters that do not copy correctly). For multiple quotations, separate each using the word AND (capitalized).	

OPEN SCIENCE BADGES

10a. Does the policy state that the journal awards one or more of the following open-science badges? Check all that apply.<br><br>"Open sciences badges" are digital badges for empirical articles that acknowledge the use of open science practices (i.e., preregistration, data sharing, and materials sharing) in the study report. Select any badges that are mentioned by name in the journal policies. See: https://cos.io/our-services/open-science-badges/	☐ No, the journal policy does not state that the journal awards badges<br>☐ "Preregistration" Badge<br>☐ "Open Data" Badge<br>☐ "Open Materials" Badge
10b. Directly copy and paste questions about OPEN SCIENCE BADGES from the submission system.<br><br>Copy entire sections that are relevant. Copy verbatim text and use quotation marks; do not otherwise edit the text (e.g., there is no need to remove line breaks or to edit characters that do not copy correctly). For multiple quotations, separate each using the word AND (capitalized).	(Citation Standards text)

Reminder

If you have completed your review for "[jn]", please remember to update the "Journal Policy Data Extraction Assignments" by changing the status column to the right of your name from "Assigned" to "Complete."<br><br>Journal Policy Data Extraction Assignments	◯ I updated the corresponding status column

Reviewer Comments

Enter any comments about using this form or questions about rating this journal's policy.
