# Supplementary material for: Evaluating implementation of the Transparency and Openness Promotion (TOP) guidelines: the TRUST process for rating journal policies, procedures, and practices
Source: Res Integr Peer Rev. 2021 Jun 2;6:9. doi: 10.1186/s41073-021-00112-8 (PMC8173977; doi:10.1186/s41073-021-00112-8)
Supplement: Supplementary file 6 — Additional file 6. [file 41073_2021_112_MOESM6_ESM.pdf]

Additional File 6: Algorithm for Determining TOP Factor Level

To determine "Data Citation" TOP Factor level

**Level 3**	1e=Yes
**Level 2**	1e=No
**Level 1**	1d=No
**Level 0**	1a=No OR<br>1c=No

To determine "Code Citation" TOP Factor level

**Level 3**	1h=Yes
**Level 2**	1h=No
**Level 1**	1g=No
**Level 0**	1b=No<br>1f=No

To determine "Data Transparency (newly collected data)" TOP Factor level

**Level 3**	2a=Yes AND 4a=Yes
**Level 2**	2a=Yes
**Level 1**	2b=Yes
**Level 0**	2b=No

To determine "Analytic Methods (code) transparency" TOP Factor level

**Level 3**	3a=Yes AND 4a=Yes
**Level 2**	3a=Yes
**Level 1**	3b=Yes
**Level 0**	3b=No

To determine "Research Materials Transparency" TOP Factor level

**Level 3**	5e=Yes
**Level 2**	5e=No
**Level 1**	5b=No OR<br>5c=No OR<br>5d=No
**Level 0**	5a=No

To determine "Design and Analysis Transparency" TOP Factor level

**Level 3**	6c=Yes
**Level 2**	6c=No
**Level 1**	6b=No
**Level 0**	6a=No

To determine “Registration of Studies” TOP Factor level

**Level 3**	7c=Yes
**Level 2**	7c=No
**Level 1**	7e=No
**Level 0**	7a=No OR<br>7b=No

To determine “Registration of Analysis Plans” TOP Factor level

**Level 3**	8d=Yes
**Level 2**	8d=No
**Level 1**	8c=No
**Level 0**	8a=No OR<br>8b=No

To determine "Replication" TOP Factor level

**Level 3**	9b=Both original and replication studies<br>OR<br>9b=Replication studies only<br>OR<br>9b=Unclear AND 9e=Yes
**Level 2**	IF NOT Level 3, THEN<br>9d=Both original and replication studies<br>OR<br>9d=Replication studies only<br>OR<br>9d=Unclear AND 9e=Yes
**Level 1**	IF NOT Level 2, THEN<br>9e=Yes
**Level 0**	IF NOT Level 1, THEN Level 0

To determine "Registered Reports & Publication BiasPublication bias" TOP Factor level

**Level 3**	9b=Both original and replication studies<br>OR<br>9b=Original studies only<br>OR<br>9b=Unclear
**Level 2**	IF NOT Level 3, THEN<br>9d=Both original and replication studies<br>OR<br>9d=Original studies only<br>OR<br>9d=Unclear
**Level 1**	IF NOT Level 2, THEN<br>9f=Yes
**Level 0**	IF NOT Level 1, THEN Level 0

To determine "Open Science Badges" TOP Factor level

**Level 3**	Not Applicable
**Level 2**	IF Journal awards all 3 open science badges
**Level 1**	IF Journal awards 1 or 2 open science badges
**Level 0**	IF Journal awards no science badges
