# Supplementary material for: Evaluating implementation of the Transparency and Openness Promotion (TOP) guidelines: the TRUST process for rating journal policies, procedures, and practices
Source: Res Integr Peer Rev. 2021 Jun 2;6:9. doi: 10.1186/s41073-021-00112-8 (PMC8173977; doi:10.1186/s41073-021-00112-8)
Supplement: Supplementary file 8 — Additional file 8. [file 41073_2021_112_MOESM8_ESM.pdf]

Additional file 8: Procedure skip logic and data codebook

	#	Variable / Field Name	Field Label<br>*Field Note*	Field Attributes (Field Type, Validation, Choices, Calculations, etc.)
Instrument: **TRUST_Procedure_Evaluation_Form** (trust_procedure_evaluation_form) Enabled as survey Collapse				
	1	record_id		text (integer, Min: 1, Max: 12)
	2	username	Please choose your name from the drop-down menu.	dropdown, Required<br>1 Rater_1_name<br>2 Rater_2_name
	3	username_confirm	[username] Please type your first name in the box (first letter in uppercase rest in lowercase).	text, Required
	4	jrnlname	JOURNAL ASSIGNMENTS: Select one of the journals for review. The journal procedure documents are located on Google Drive. Please make sure not to edit or remove any of the documents in this folder. Below is the link to the journal procedure documents.<br>[redacted]	dropdown (autocomplete), Required<br>1 Journal_names
	5	jrnlname_confirm	[journal name] Please type name of the selected journal in the box (first letter in uppercase rest in lowercase).	text, Required
	6	sub_system	SUBMISSION SYSTEM: Please select the submission sytem used by the journal you are rating from the drop down menu.	dropdown, Required<br>1 ScholarOne<br>2 Editorial Manager<br>3 EVISE<br>4 Elsevier Editorial System<br>5 Other
	7	other_sub_system<br>Show the field ONLY if:<br>[sub_system] = '5'	Please type name of the other submission system in the text box	text, Required
	8	pro_1a_data_citation	Section Header: *DATA CITATION*<br>1a. Does the submission system ask authors to confirm that data have been cited according to journal guidelines? Answer "Yes" if the submission system asks authors to confirm that they have included in their reference list or bibliography a citation to the dataset(s) underlying the results reported in their study. Answer "No" the submission system references publisher guidelines or a standalone style manual but does not explicitly mention citation of datasets.	yesno, Required<br>1 Yes<br>0 No
	9	pro_1b_data_cit_confirm<br>Show the field ONLY if:<br>[pro_1a_data_citation] = '1'	1b. Are authors required to confirm that data have been cited according to journal guidelines in order to proceed (i.e., an answer is required by the submission system)? Answer "Yes" if this information is required for one or more types of studies (e.g., clinical trials). For example, the field might appear for only certain study types.	yesno, Required<br>1 Yes<br>0 No
	10	pro_1c_data_cit_text	1c. Directly copy and paste questions about DATA CITATION from the submission system. Copy verbatim text and use quotation marks; do not otherwise edit the text (e.g., there is no need to remove line breaks or to edit characters that do not copy correctly). For multiple quotations, separate each using the word AND (capitalized).	notes

	11	pro_2a_data_link	Section Header: *DATA TRANSPARENCY*<br>2a. Does the submission process include one or more fields in which authors may enter a link to DATA (i.e., DOI, URL, or other persistent identifier) ? Answer “Yes” if the submission process includes a field for providing a link to a dataset, even if the field is not required. Answer “Yes” if this information may be entered for one or more types of studies (e.g., clinical trials). Answer “No” if the system indicates that this information should be included in the manuscript file or another attachment, but does not include a field to enter this information in the submission system itself. Answer “No” if the field in which authors may enter a link to DATA (i.e., DOI, URL, or other persistent identifier) is not specific to DATA ONLY.	yesno, Required<br>1 Yes<br>0 No
	12	pro_2b_req_data_link<br>Show the field ONLY if:<br>[pro_2a_data_link] = '1'	2b. Are authors required to enter a link to DATA in order to proceed (i.e., is the field required)? Answer “Yes” if this information is required for one or more types of studies (e.g., clinical trials). For example, the field might appear for only certain study types.	yesno, Required<br>1 Yes<br>0 No
	13	pro_2c_data_pub	2c. Does the submission process include one or more fields for indicating whether DATA are publicly available? Answer “Yes” if the submission process includes a “Yes/No” question (e.g., using radio buttons). Answer “Yes” if the submission process includes a field for a “data availability” or “data access” statement. Still answer “Yes” if this field includes more than “empirical data underlying reported results” in its definition “data”, so long as “empirical data underlying reported results” is included in this expanded definition (e.g., if the definition of “data” includes raw data, processed data, software, algorithms, protocols, methods, and materials). However, answer “No” if there is a RADIO BUTTON, CHECKBOX, or DROP-DROWN MENU for “indicating whether data are publicly available”, and it includes more than “empirical data underlying reported results” in its definition of “data” (e.g., if the definition of “data” includes software, algorithms, protocols, methods, and materials) Answer “Yes” if this information is requested for one or more types of studies (e.g., clinical trials).	yesno, Required<br>1 Yes<br>0 No
	14	pro_2d_data_req_to_ind<br>Show the field ONLY if:<br>[pro_2c_data_pub] = '1'	2d. Are authors required to indicate whether the DATA are available in order to proceed (i.e., is the field required)? Answer “Yes” if this information is required for one or more types of studies (e.g., clinical trials).	yesno, Required<br>1 Yes<br>0 No
	15	pro_2e_data_incld_field	2e. Does the submission process include one or more fields for authors to upload their DATA? Answer “Yes” if the submission process includes a field specifically for uploading a dataset, even if the field is not required. Answer “No” if the system only has a field for uploading “supplementary materials” generally with no explicit mention of uploading datasets. Answer “No” if the field for “uploading data” or “sharing data” includes more than “empirical data underlying reported results” in its definition “data” (e.g., if the definition of “data” includes software, algorithms, protocols, methods, and materials). Answer “No” if the field in which authors may upload DATA is not specific to DATA ONLY.	yesno, Required<br>1 Yes<br>0 No
	16	pro_2f_req_upload<br>Show the field ONLY if:<br>[pro_2e_data_incld_field] = '1'	2f. Are authors required to upload their DATA in order to proceed (i.e., is the field required)?	yesno, Required<br>1 Yes<br>0 No
	17	pro_2g_data_trans_text	2g. Directly copy and paste questions about DATA TRANSPARENCY from the submission system. Copy verbatim text and use quotation marks; do not otherwise edit the text (e.g., there is no need to remove line breaks or to edit characters that do not copy correctly). For multiple quotations, separate each using the word AND (capitalized).	notes

	18	pro_3a_code_link	Section Header: *ANALYTIC METHODS (CODE) TRANSPARENCY*<br>3a. Does the submission process include one or more fields in which authors may enter a link to CODE (i.e., DOI, URL, or other persistent identifier) posted on a repository? Repositories are permanent collections such as FigShare, Dryad, and institutional repositories. Answer “Yes” if the submission process includes a field for providing a link to code, even if the field is not required. Answer “Yes” if this information may be entered for one or more types of studies (e.g., clinical trials). Answer “No” if the system indicates that this information should be included in the manuscript file or another attachment, but does not include a field to enter this information in the submission system itself. Answer “No” if the field in which authors may enter a link to CODE (i.e., DOI, URL, or other persistent identifier) posted on a repository is not specific to CODE ONLY.	yesno, Required<br>1 Yes<br>0 No
	19	pro_3b_code_req_link<br>Show the field ONLY if:<br>[pro_3a_code_link] = '1'	3b. Are authors required to enter a link to CODE in order to proceed (i.e., is the field required)? Answer “Yes” if this information is required for one or more types of studies (e.g., clinical trials). For example, the field might appear for only certain study types.	yesno, Required<br>1 Yes<br>0 No
	20	pro_3c_code_public	3c. Does the submission process include one or more fields for indicating whether CODE are publicly available? Answer “Yes” if the submission process includes a “Yes/No” question (e.g., using radio buttons). Answer “Yes” if the submission process includes a field for a “code availability” or “code access” statement. Answer “Yes” if the submission process includes a field for a “data availability” or “data access” statement if (and only if) this field explicitly includes “code” in its definition “data”. Answer “Yes” if this information is requested for one or more types of studies (e.g., clinical trials). Answer “No” if there is a RADIO BUTTON, CHECKBOX, or DROP-DROWN MENU for “indicating whether data are publicly available”, and it includes more than “empirical data underlying reported results” in its definition of “data” (e.g., if the definition of “data” includes software, algorithms, protocols, methods, and materials), even if this definition includes code.	yesno, Required<br>1 Yes<br>0 No
	21	pro_3d_code_req_to_ind<br>Show the field ONLY if:<br>[pro_3c_code_public] = '1'	3d. Are authors required to indicate whether the CODE are available in order to proceed (i.e., is the field required)? Answer “Yes” if this information is required for one or more types of studies (e.g., clinical trials).	yesno, Required<br>1 Yes<br>0 No
	22	pro_3e_code_field	3e. Does the submission process include one or more fields for authors to upload their CODE? Answer “Yes” if the submission process includes a field specifically for uploading code, even if the field is not required. Answer “No” if the system only has a field for uploading “supplementary materials” generally with no explicit mention of uploading code. Answer “No” if the field in which authors may upload CODE is not specific to CODE ONLY.	yesno, Required<br>1 Yes<br>0 No
	23	pro_3f_code_req_upload<br>Show the field ONLY if:<br>[pro_3e_code_field] = '1'	3f. Are authors required to upload their CODE in order to proceed (i.e., is the field required)?	yesno, Required<br>1 Yes<br>0 No
	24	pro_3g_code_text	3g. Directly copy and paste questions about ANALYTIC METHODS (CODE) TRANSPARENCY from the submission system. Copy verbatim text and use quotation marks; do not otherwise edit the text (e.g., there is no need to remove line breaks or to edit characters that do not copy correctly). For multiple quotations, separate each using the word AND (capitalized).	notes

	25	pro_4a_mtrls_link	Section Header: *RESEARCH MATERIALS TRANSPARENCY*<br>4a. Does the submission process include one or more fields in which authors may enter a link to newly created RESEARCH MATERIALS (i.e., DOI, URL, or other persistent identifier) posted on a repository? Research materials refer to specific items that would be necessary for others to conduct an independent replication of the research, such as those used in the intervention (e.g. instructional materials, proprietary computer programs, etc.) or for data collection (e.g. questionnaires, interview guides). Consider only materials created for the current study; do not consider commercially available tools (e.g., statistical analysis programs) or materials that are being re-used from previous studies. Answer "Yes" if the submission process includes a field for providing a link to research materials, even if the field is not required. Answer "Yes" if this information may be entered for one or more types of studies (e.g., clinical trials). Answer "No" if the system indicates that this information should be included in the manuscript file or another attachment, but does not include a field to enter this information in the submission system itself. Answer "No" if the fields in which authors may enter a link to newly created RESEARCH MATERIALS (i.e., DOI, URL, or other persistent identifier) posted on a repository is not specific to RESEARCH MATERIALS only.	yesno, Required<br>1 Yes<br>0 No
	26	pro_4b_mtrls_req_link<br>Show the field ONLY if:<br>[pro_4a_mtrls_link] = '1'	4b. Are authors required to enter a link to RESEARCH MATERIALS in order to proceed (i.e., is the field required)? Answer "Yes" if this information is required for one or more types of studies (e.g., clinical trials). For example, the field might appear for only certain study types.	yesno, Required<br>1 Yes<br>0 No
	27	pro_4c_mtrls_pub	4c. Does the submission process include one or more fields for indicating whether RESEARCH MATERIALS are publicly available? Answer "Yes" if the submission process includes a "Yes/No" question (e.g., using radio buttons). Answer "Yes" if the submission process includes a field for a "research materials availability" or "research materials access" statement. Answer "Yes" if the submission process includes a field for a "data availability" or "data access" statement if (and only if) this field explicitly includes "research materials" in its definition "data". Answer "Yes" if this information is requested for one or more types of studies (e.g., clinical trials). Answer "No" if there is a RADIO BUTTON, CHECKBOX, or DROP-DROWN MENU for "indicating whether data are publicly available", and it includes more than "empirical data underlying reported results" in its definition of "data" (e.g., if the definition of "data" includes software, algorithms, protocols, methods, and materials), even if this definition includes research materials.	yesno, Required<br>1 Yes<br>0 No
	28	pro_4d_mtrls_req_to_ind<br>Show the field ONLY if:<br>[pro_4c_mtrls_pub] = '1'	4d. Are authors required to indicate whether the RESEARCH MATERIALS are available in order to proceed (i.e., is the field required)? Answer "Yes" if this information is required for one or more types of studies (e.g., clinical trials).	yesno, Required<br>1 Yes<br>0 No
	29	pro_4e_mtrls_incld_field	4e. Does the submission process include one or more fields for authors to upload their RESEARCH MATERIALS? Answer "Yes" if the submission process includes a field specifically for uploading research materials, even if the field is not required. Answer "No" if the system only has a field for uploading "supplementary materials" generally with no explicit mention of uploading research materials. Answer "No" if the field in which authors may upload RESEARCH MATERIALS is not specific to RESEARCH MATERIALS ONLY.	yesno, Required<br>1 Yes<br>0 No
	30	pro_4f_mtrls_req_upload<br>Show the field ONLY if:<br>[pro_4e_mtrls_incld_field] = '1'	4f. Are authors required to upload their RESEARCH MATERIALS in order to proceed (i.e., is the field required)?	yesno, Required<br>1 Yes<br>0 No

	31	pro_4g_mtrls_text	4g. Directly copy and paste questions about RESEARCH MATERIALS TRANSPARENCY from the submission system. Copy verbatim text and use quotation marks; do not otherwise edit the text (e.g., there is no need to remove line breaks or to edit characters that do not copy correctly). For multiple quotations, separate each using the word AND (capitalized).	notes
	32	pro_5a_gl_checklist	Section Header: *DESIGN AND ANALYSIS TRANSPARENCY*<br>5a. Does the submission process include a field for uploading a completed checklist for one or more reporting guidelines (e.g., a CONSORT checklist)? Reporting guidelines describe the minimum information about study methods and results that should be included in a journal article. Reporting guidelines differ from “style guides” that describe how information should be reported (rather than what information to report). Most reporting guidelines include a checklist and a flow diagram and have an acronym or name (e.g., “JARS”). There may not be an appropriate reporting guideline for some studies. Answer “Yes” if the system has a field for uploading a completed checklist with a quotation or page number corresponding to each item in the reporting guideline. Answer “Yes” if this information is requested for one or more types of studies (e.g., clinical trials). Answer “No” if the system indicates that reports must include complete information about their methods and results, but does not include a field to enter this information in the submission system itself.	yesno, Required<br>1 Yes<br>0 No
	33	pro_5b_chklst_req<br>Show the field ONLY if: [pro_5a_gl_checklist] = '1'	5b. Are authors required to upload a completed checklist for one or more reporting guidelines in order to proceed (i.e., is the field required)? Answer “Yes” if this information is required for one or more types of studies (e.g., clinical trials).	yesno, Required<br>1 Yes<br>0 No
	34	pro_5c_ind_reporting	5c. Does the submission process include a field for indicating whether the authors followed a reporting guideline (e.g., by reference to the EQUATOR Network, CONSORT, or a checklist of specific items used by the journal)? Answer “Yes” if the submission process includes a “Yes/No” question (e.g., using radio buttons). Answer “Yes” if this information is requested for one or more types of studies (e.g., clinical trials). Answer “No” if authors are required to upload a form that includes an item for indicating whether the authors followed a reporting guideline, but the submission system itself does not have an explicit and dedicated field for indicating whether the authors followed a reporting guideline.	yesno, Required<br>1 Yes<br>0 No
	35	pro_5d_gl_req_to_ind<br>Show the field ONLY if: [pro_5c_ind_reporting] = '1'	5d. Are authors required to indicate whether they followed a reporting guideline in order to proceed (i.e., is the field required)? Answer “Yes” if this information is required for one or more types of studies (e.g., clinical trials).	yesno, Required<br>1 Yes<br>0 No
	36	pro_5e_link_proto	5e. Does the submission process include a field for linking to or uploading a study protocol? A protocol is a document that details the rationale, proposed methods, organisation, and ethical considerations of a study. Answer “Yes” if this information is requested for one or more types of studies (e.g., clinical trials). Answer “No” if the field for linking to or uploading a study protocol is not specific to STUDY PROTOCOL only.	yesno, Required<br>1 Yes<br>0 No
	37	pro_5f_req_to_link<br>Show the field ONLY if: [pro_5e_link_proto] = '1'	5f. Are authors required to link to or upload a protocol in order to proceed (i.e., is the field required)? Answer “Yes” if this information is required for one or more types of studies (e.g., clinical trials).	yesno, Required<br>1 Yes<br>0 No

	38	pro_5g_proto_avail	5g. Does the submission process include a field for indicating whether the study protocol is available? Answer “Yes” if the submission process includes a “Yes/No” question (e.g., using radio buttons). Answer “Yes” if the submission process includes a field for a “research materials availability” or “research materials access” statement. Answer “Yes” if this information is requested for one or more types of studies (e.g., clinical trials). Answer “No” if there is a RADIO BUTTON, CHECKBOX, or DROP-DOWN MENU for “indicating whether data are publicly available”, and it includes more than “empirical data underlying reported results” in its definition of “data” (e.g., if the definition of “data” includes software, algorithms, protocols, methods, and materials), even if this definition includes study protocols.	yesno, Required<br>1 Yes<br>0 No
	39	pro_5h_proto_req_to_ind<br>Show the field ONLY if: [pro_5g_proto_avail] = '1'	5h. Are authors required to indicate whether a protocol is available in order to proceed (i.e., is the field required)? Answer “Yes” if this information is required for one or more types of studies (e.g., clinical trials).	yesno, Required<br>1 Yes<br>0 No
	40	pro_5i_design_text	5i. Directly copy and paste questions about DESIGN AND ANALYSIS from the submission system. Copy verbatim text and use quotation marks; do not otherwise edit the text (e.g., there is no need to remove line breaks or to edit characters that do not copy correctly). For multiple quotations, separate each using the word AND (capitalized).	notes
	41	pro_6a_study_reg	Section Header: *REGISTRATION OF STUDIES*<br>6a. Does the submission process include one or more fields in which authors may enter a study registration number or a link to the STUDY REGISTRATION (i.e., URL or DOI)? Answer “Yes” if the submission process includes a field for providing a link or a study registration number, even if the field is not required. Answer “Yes” if this information may be entered for one or more types of studies (e.g., clinical trials). Answer “No” if the system indicates that this information should be included in the manuscript file or another attachment, but does not include a field to enter this information in the submission system itself.	yesno, Required<br>1 Yes<br>0 No
	42	pro_6b_req_reg<br>Show the field ONLY if: [pro_6a_study_reg] = '1'	6b. Are authors required to enter a STUDY REGISTRATION number or a link to to the study registration (i.e., is the field required)? Answer “Yes” if this information is required for one or more types of studies (e.g., clinical trials). For example, the field might appear for only certain study types.	yesno, Required<br>1 Yes<br>0 No
	43	pro_6c_incl_reg	6c. Does the submission process include a field for indicating whether the study was registered? Answer “Yes” if the submission process includes a “Yes/No” question (e.g., using radio buttons). Answer “Yes” if this information is requested for one or more types of studies (e.g., clinical trials). Answer “No” if authors are required to upload a form that includes an item for indicating whether the study was registered, but the submission system itself does not have an explicit and dedicated field for indicating whether the study was registered.	yesno, Required<br>1 Yes<br>0 No
	44	pro_6d_req_to_ind<br>Show the field ONLY if: [pro_6c_incl_reg] = '1'	6d. Are authors required to indicate whether the study was registered in order to proceed (i.e., is the field required)? Answer “Yes” if this information is required for one or more types of studies (e.g., clinical trials).	yesno, Required<br>1 Yes<br>0 No

	45	pro_6e_prspt_reg<br><br>Show the field ONLY if:<br>[pro_6a_study_reg] = '1' or [pro_6c_incl_reg] = '1'	6e. Does the submission process include a field for indicating whether the study was registered PROSPECTIVELY? “Prospective” registration occurs before a key event, such as before enrolling the first participant, before completing data collection, or before analyzing the data. Answer “Yes” if there is one question about registration and it includes information about whether the study was registered prospectively. Answer “Yes” if the submission system includes a separate field related to “prospective registration” or “preregistration”, or if the submission system includes a field for indicating whether studies were registered before beginning or completing enrollment, or before completing data collection. Answer “Yes” if this information is requested for one or more types of studies (e.g., clinical trials).	yesno, Required<br>1 Yes<br>0 No
	46	pro_6f_prspt_req_to_ind<br><br>Show the field ONLY if:<br>[pro_6e_prspt_reg] = '1'	6f. Are authors required to indicate whether the study was registered PROSPECTIVELY in order to proceed (i.e., is the field required)? Answer “Yes” if this information is required for one or more types of studies (e.g., clinical trials).	yesno, Required<br>1 Yes<br>0 No
	47	pro_6g_reg_text	6g. Directly copy and paste questions about REGISTRATION OF STUDIES from the submission system. Copy verbatim text and use quotation marks; do not otherwise edit the text (e.g., there is no need to remove line breaks or to edit characters that do not copy correctly). For multiple quotations, separate each using the word AND (capitalized).	notes
	48	pro_7a_analysis_reg	Section Header: *REGISTRATION OF ANALYSIS PLANS*<br><br>7a. Does the submission process include one or more fields in which authors may enter a location or a link to the ANALYSIS PLAN (i.e., URL or DOI)? Answer “Yes” if the submission process includes a field for providing a location or link to an analysis plan, even if the field is not required. Answer “Yes” if this information may be entered for one or more types of studies (e.g., clinical trials). Answer “No” if the system indicates that this information should be included in the manuscript file or another attachment, but does not include a field to enter this information in the submission system itself.	yesno, Required<br>1 Yes<br>0 No
	49	pro_7b_analysis_link<br><br>Show the field ONLY if:<br>[pro_7a_analysis_reg] = '1'	7b. Are authors required to enter a location or a link to the ANALYSIS PLAN (i.e., is the field required)? Answer “Yes” if this information is required for one or more types of studies (e.g., clinical trials). For example, the field might appear for only certain study types.	yesno, Required<br>1 Yes<br>0 No
	50	pro_7c_ind_analy_reg	7c. Does the submission process include a field for indicating whether the ANALYSIS PLAN was registered? Answer “Yes” if the submission process includes a “Yes/No” question (e.g., using radio buttons). Answer “Yes” if this information is requested for one or more types of studies (e.g., clinical trials). Answer “No” if authors are required to upload a form that includes an item for indicating whether the analysis plan was registered, but the submission system itself does not have an explicit and dedicated field for indicating whether the analysis plan was registered.	yesno, Required<br>1 Yes<br>0 No
	51	pro_7d_analy_req_toind<br><br>Show the field ONLY if:<br>[pro_7c_ind_analy_reg] = '1'	7d. Are authors required to indicate whether the ANALYSIS PLAN was registered in order to proceed (i.e., is the field required)? Answer “Yes” if this information is required for one or more types of studies (e.g., clinical trials).	yesno, Required<br>1 Yes<br>0 No

	52	pro_7e_ind_prspt_reg<br><br>Show the field ONLY if:<br>[pro_7a_analysis_reg] = '1' or [pro_7c_ind_analy_reg] = '1'	7e. Does the submission process include a field for indicating whether the ANALYSIS PLAN was registered PROSPECTIVELY? “Prospective” registration occurs before a key event, such as before enrolling the first participant, before completing data collection, or before analyzing the data. Answer “Yes” if there is one question about registration and it includes information about whether the analysis plan was registered prospectively. Answer “Yes” if the submission system includes a separate field related to “prospective registration” or “preregistration”, or if the submission system includes a field for indicating whether studies were registered before beginning or completing enrollment, or before completing data collection. Answer “Yes” if this information is requested for one or more types of studies (e.g., clinical trials).	yesno, Required<br>1 Yes<br>0 No
	53	pro_7f_req_to_proceed<br><br>Show the field ONLY if:<br>[pro_7e_ind_prspt_reg] = '1'	7f. Are authors required to indicate whether the ANALYSIS PLAN was registered PROSPECTIVELY in order to proceed (i.e., is the field required)? Answer “Yes” if this information is required for one or more types of studies (e.g., clinical trials).	yesno, Required<br>1 Yes<br>0 No
	54	pro_7g_upload_field	7g. Does the submission process include a field for uploading an analysis plan? Answer “Yes” if there is a field for uploading a “statistical analysis plan” or “SAP” as supplementary material. Answer “Yes” if this information is requested for one or more types of studies (e.g., clinical trials).	yesno, Required<br>1 Yes<br>0 No
	55	pro_7h_req_to_upload<br><br>Show the field ONLY if:<br>[pro_7g_upload_field] = '1'	7h. Are authors required to upload an analysis plan in order to proceed (i.e., is the field required)? Answer “Yes” if this information is required for one or more types of studies (e.g., clinical trials).	yesno, Required<br>1 Yes<br>0 No
	56	pro_7i_analy_reg_text	7i. Directly copy and paste questions about REGISTRATION OF ANALYSIS PLAN from the submission system. Copy verbatim text and use quotation marks; do not otherwise edit the text (e.g., there is no need to remove line breaks or to edit characters that do not copy correctly). For multiple quotations, separate each using the word AND (capitalized).	notes
	57	pro_8a_ind_repl	Section Header: *REPLICATION*<br><br>8a. Does the submission process include a field for indicating whether the study is a replication? “Replication” studies aim to reproduce the methods and results of previous studies, and are typically described using the term “replication”.	yesno, Required<br>1 Yes<br>0 No
	58	pro_8b_repl_req_toind<br><br>Show the field ONLY if:<br>[pro_8a_ind_repl] = '1'	8b. Are authors required to indicate whether the study is a replication in order to proceed (i.e., is the field required)? Answer “Yes” if this information is required for one or more types of studies (e.g., clinical trials).	yesno, Required<br>1 Yes<br>0 No
	59	pro_8c_blind_rev<br><br>Show the field ONLY if:<br>[pro_8a_ind_repl] = '1'	Results-Blind Review 8c. Does the submission process include a field for indicating whether authors are submitting a replication study for results blind review?	yesno, Required<br>1 Yes<br>0 No
	60	pro_8d_blind_req_ind<br><br>Show the field ONLY if:<br>[pro_8c_blind_rev] = '1'	8d. Are authors required to indicate whether the study being submitted is a replication for results blind review in order to proceed (i.e., is the field required)? Answer “Yes” if this information is required for one or more types of studies (e.g., clinical trials).	yesno, Required<br>1 Yes<br>0 No
	61	pro_8e_reg_rep_field<br><br>Show the field ONLY if:<br>[pro_8a_ind_repl] = '1'	Registered Replication Reports 8e. Does the submission process include a field for indicating whether authors are submitting a replication study as a “Registered Report”?	yesno, Required<br>1 Yes<br>0 No
	62	pro_8f_reg_rep_req2ind<br><br>Show the field ONLY if:<br>[pro_8e_reg_rep_field] = '1'	8f. Are authors required to indicate whether the study being submitted is a “Registered Replication Report” in order to proceed (i.e., is the field required)? Answer “Yes” if this information is required for one or more types of studies (e.g., clinical trials).	yesno, Required<br>1 Yes<br>0 No

	63	pro_8g_repl_text	8g. Directly copy and paste questions about REPLICATION from the submission system. Copy verbatim text and use quotation marks; do not otherwise edit the text (e.g., there is no need to remove line breaks or to edit characters that do not copy correctly). For multiple quotations, separate each using the word AND (capitalized).	notes
	64	pro_9a_out_rep	Section Header: *PUBLICATION BIAS (Original Research)*<br>Significance/novelty 9a. Does the submission system ask authors to affirm that all outcomes have been reported, regardless of significance or novelty of findings? Answer "Yes" if the submission includes a transparency declaration asking the authors to affirm that this manuscript is an honest, accurate, comprehensive account of the study being reported (e.g., affirming that no outcomes have not been reported based on the nature of the findings).	yesno, Required<br>1 Yes<br>0 No
	65	pro_9b_req_out_rep<br>Show the field ONLY if: [pro_9a_out_rep] = '1'	9b. Are authors required to affirm that all outcomes have been reported, regardless of significance or novelty of findings? Answer "Yes" if this information is required for one or more types of studies (e.g., clinical trials).	yesno, Required<br>1 Yes<br>0 No
	66	pro_9c_field_brev	Results-Blind Review 9c. Does the submission process include a field for indicating whether the study is being submitted for results blind review?	yesno, Required<br>1 Yes<br>0 No
	67	pro_9d_brev_req2ind<br>Show the field ONLY if: [pro_9c_field_brev] = '1'	9d. Are authors required to indicate whether the study is being submitted for results blind review in order to proceed (i.e., is the field required)? Answer "Yes" if this information is required for one or more types of studies (e.g., clinical trials).	yesno, Required<br>1 Yes<br>0 No
	68	pro_9e_reg_rep	Registered Reports 9e. Does the submission process include a field for indicating whether the study is a "Registered Report"?	yesno, Required<br>1 Yes<br>0 No
	69	pro_9f_reg_rep_req2ind<br>Show the field ONLY if: [pro_9e_reg_rep] = '1'	9f. Are authors required to indicate whether the study is a "Registered Report" in order to proceed (i.e., is the field required)? Answer "Yes" if this information is required for one or more types of studies (e.g., clinical trials).	yesno, Required<br>1 Yes<br>0 No
	70	pro_9g_pub_bias_text	9g. Directly copy and paste questions about PUBLICATION BIAS from the submission system. Copy verbatim text and use quotation marks; do not otherwise edit the text (e.g., there is no need to remove line breaks or to edit characters that do not copy correctly).	notes
	71	pro_10a_prereg_badg	Section Header: *OPEN SCIENCE BADGES*<br>Preregistered Badge 10a. Does the submission system ask authors whether they wish to be considered for the "Pre-registered" badge? Answer "Yes" if the submission system asks authors to affirm or provide a statement (e.g., in an "Open Practices Disclosure Form") that they achieved the criteria for a digital badge accompanying the article to signify that they pre-registered their study.).	yesno, Required<br>1 Yes<br>0 No
	72	pro_10b_wish_badge<br>Show the field ONLY if: [pro_10a_prereg_badg] = '1'	10b. Are authors required to indicate whether they wish to be considered for the "Preregistered" badge? Answer "Yes" if this information is required for one or more types of studies (e.g., clinical trials).	yesno, Required<br>1 Yes<br>0 No
	73	pro_10c_open_data	Open Data Badge 10c. Does the submission system ask authors whether they wish to be considered for the "Open Data" badge? Answer "Yes" if the submission system asks authors to affirm or provide a statement (e.g., in an "Open Practices Disclosure Form") that they achieved the criteria for a digital badge accompanying the article to signify that they have shared their data in a repository.).	yesno, Required<br>1 Yes<br>0 No
	74	pro_10d_req2ind_open_data<br>Show the field ONLY if: [pro_10c_open_data] = '1'	10d. Are authors required to indicate whether they wish to be considered for the "Open Data" badge? Answer "Yes" if this information is required for one or more types of studies (e.g., clinical trials).	yesno, Required<br>1 Yes<br>0 No

	75	pro_10e_open_mtrls	Open Materials Badge 10e. Does the submission system ask authors whether they wish to be considered for the "Open Materials" badge? Answer "Yes" if the submission system asks authors to affirm or provide a statement (e.g., in an "Open Practices Disclosure Form") that they achieved the criteria for a digital badge accompanying the article to signify that they have shared their materials in a repository.).	yesno, Required<br>1 Yes<br>0 No
	76	pro_10f_req2ind_open_mtrls<br>Show the field ONLY if:<br>[pro_10e_open_mtrls] = '1'	10f. Are authors required to indicate whether they wish to be considered for the "Open Materials" badge? Answer "Yes" if this information is required for one or more types of studies (e.g., clinical trials).ared their materials in a repository.	yesno, Required<br>1 Yes<br>0 No
	77	pro_10g_open_sci_text	10g. Directly copy and paste questions about OPEN SCIENCE BADGES from the submission system. Copy verbatim text and use quotation marks; do not otherwise edit the text (e.g., there is no need to remove line breaks or to edit characters that do not copy correctly).	notes
	78	verify_assign_sheet	Please check the box after changing "Rater status" in procedure assignment sheet (link below) from "Assigned" to "Complete".<br>[redacted]	checkbox<br>1 verify_assign_sheet___1 Completed
	79	comment_box_2	Enter any comments about using this form or questions about rating this journal's procedure.	notes
	80	trust_procedure_evaluation_form_complete	Section Header: *Form Status*<br>Complete?	dropdown<br>0 Incomplete<br>1 Unverified<br>2 Complete
