# Supplementary material for: Evaluating implementation of the Transparency and Openness Promotion (TOP) guidelines: the TRUST process for rating journal policies, procedures, and practices
Source: Res Integr Peer Rev. 2021 Jun 2;6:9. doi: 10.1186/s41073-021-00112-8 (PMC8173977; doi:10.1186/s41073-021-00112-8)
Supplement: Supplementary file 10 — Additional file 10. [file 41073_2021_112_MOESM10_ESM.pdf]

Additional file 10: Practices Evaluation Tool
Revised January 5, 2021

This instrument is designed to rate published journal article concordance with TOP standards. It uses a series of factual questions to indicate whether journal policies and procedures lead to authors providing information related to TOP in published articles (i.e., journal practice). For any given practice, the instrument should be completed by at least two independent raters.

DESCRIPTIVE INFORMATION

Did the authors of the study collect the data? [Don't copy and paste the whole methods section. Include page numbers (for the paginated article, not the PDF). Answer "Yes" if the journal article reports results for a study that the authors conducted using data that they collected (e.g., as in an article reporting the main results of a randomized trial). Answer "No" if the journal article reports results for a study that the authors conducted using data that were collected previously (e.g., a publicly available cohort, data collected routinely by the government). Answer "Yes" if the study involves both primary and secondary data. Include quotations that imply data were collected "we conducted a RCT" or, if necessary, a note.]

Yes

No

1) DATA CITATION

1a. Is the dataset used in the study cited in the text of the article as an in-text citation using the same format as citations for other journal articles and books, e.g., (Mayo-Wilson & Grant, 2020)? [Answer "Yes" if the journal article includes a citation to the dataset in the main text. Answer "No" if the main narrative mentions the dataset but does not provide a citation. Answer "No" if the manuscript includes a URL or other link to the data but the manuscript does not include a formal in-text citation.]

Yes

No

1b. Is the dataset used in the study cited in the references section? [Answer "Yes" if the journal article includes a citation to the dataset in the bibliography/references section using the same format as citations for other journal articles and books. Answer "No" if the article mentions the dataset but does not provide a formal citation in the bibliography/references section.].

Yes

No

1c. [Display IF 1b=Yes] In the bibliography/references section, does the citation to the dataset used in the study include a persistent identifier? [Answer "Yes" if the citation includes a persistent identifier such as a "Digital Object Identifier" ("DOI") for the location of the dataset. Answer "Yes" if the citation includes a URL (website) for a trusted repository; however, answer "No" if the citation includes a URL to a website other than a trusted repository. Trusted repositories are permanent, independently-controlled collections that are accessible to the public, such as FigShare, Dryad, and university/institutional repositories. Answer "No" if the citation refers to an article or other resource that merely describes that dataset.]

Yes

No

1d. Directly copy and paste questions about DATA CITATION from the journal article. [Copy verbatim text and use quotation marks; do not otherwise edit the text (e.g., there is no need to remove line breaks or to edit characters that do not copy correctly). For multiple quotations, separate each using the word AND (capitalized).]

2) DATA, CODE, AND RESEARCH MATERIALS TRANSPARENCY

2a. Does the article include __a designated section__ with a statement about the availability of the data underlying the findings reported in the article? [An “availability statement” need not have the specific heading (e.g., “Data transparency statement”), but should contain information about transparency be in a dedicated section in the abstract, beginning or the article, or end of the article. Answer “Yes” if the journal article includes a “data availability”, “data access”, or “data sharing” statement in a footnote, endnote, or a section of acknowledgements or declarations following the main text. Answer “No” if the main text of the journal article includes a statement about whether or not the data underlying the reported findings are publicly available but a statement does not appear in one of the sections listed above. Answer “No” if any mention of data availability is not in a dedicated field within the manuscript.]

Yes

No

2b. [Display IF 2a=Yes] Does the availability statement include a persistent identifier for the dataset used in the study? [Answer “Yes” if the data availability statement includes a persistent identifier such as a "Digital Object Identifier" ("DOI") for the location of the dataset. Answer “Yes” if the statement includes a URL (website) for a trusted repository; however, answer “No” if the citation includes a URL to a website other than a trusted repository. Trusted repositories are permanent, independently-controlled collections that are accessible to the public, such as FigShare, Dryad, and university/institutional repositories. Answer “Yes” if the data availability statement includes a citation to the bibliography/references section and that citation includes the location of the dataset. Answer “No” if the citation refers to an article or other resource that merely describes the dataset.]

Yes

No

2c. Does the article include a __designated section__ with a statement about the availability of the analysis code or software used to generate the findings reported in the article?
[An “availability statement” need not have the specific heading (e.g., “Code transparency statement”), but should contain information about transparency be in a dedicated section in the abstract, beginning or the article, or end of the article. Answer “Yes” if the journal article includes a “code availability”, “code access”, or “code sharing” statement in a footnote, endnote, or a section of acknowledgements or declarations following the main text. Answer “No” if the main text of the journal article includes a statement about whether or not the code used to generate the reported findings is publicly available but a statement does not appear in one of the sections listed above. Answer “No” if any mention of code availability is not in a dedicated field within the manuscript. Answer “No” if the study does NOT include an availability statement.]

Yes

No

2d. [Display IF 2c=Yes] Does the availability statement include a persistent identifier for the code used in the study? [Answer "Yes" if the availability statement includes a persistent identifier such as a "Digital Object Identifier" ("DOI") for the location of the code. Answer "Yes" if the statement includes a URL (website) for a trusted repository; however, answer "No" if the citation includes a URL to a website other than a trusted repository. Trusted repositories are permanent, independently-controlled collections that are accessible to the public, such as FigShare, Dryad, and university/institutional repositories. Answer "Yes" if the availability statement includes a citation to the bibliography/references section and that citation includes the location of the code. Answer "No" if the citation refers to an article or other resource that merely describes the code.]

Yes

No

2e. Does the article include a <u>designated section</u> with a statement about the availability of the research materials used to conduct the study reported in the article?
[An "availability statement" need not have the specific heading (e.g., "Data transparency statement"), but should contain information about transparency be in a dedicated section in the abstract, beginning or the article, or end of the article. Answer "Yes" if the journal article includes a "research materials availability", "research materials access", or "research materials sharing" statement in a footnote, endnote, or a section of acknowledgements or declarations following the main text. Answer "No" if the main text of the journal article includes a statement about whether or not the research materials used to conduct the study reported in the article is publicly available but a statement does not appear in one of the sections listed above. Answer "No" if any mention of materials availability is not in a dedicated field within the manuscript. Answer "No" if the study does NOT include an availability statement.]

Yes

No

2f. [Display IF 2e=Yes] Does the availability statement include a persistent identifier or <u>other unique identifier</u> for the research materials used in the study? [Answer "Yes" if the statement includes a unique identifier such as an ISSN. Answer "Yes" if the availability statement includes a persistent identifier such as a "Digital Object Identifier" ("DOI") for the location of the research materials. Answer "Yes" if the statement includes a URL (website) for a trusted repository; however, answer "No" if the citation includes a URL to a website other than a trusted repository. Trusted repositories are permanent, independently-controlled collections that are accessible to the public, such as FigShare, Dryad, and university/institutional repositories. Answer "Yes" if the availability statement includes a citation to the bibliography/references section and that citation

includes the location of the research materials. Answer “No” if the citation refers to an article or other resource that merely describes the research materials.]

Yes

No

2g. Directly copy and paste questions about DATA, CODE, AND RESEARCH MATERIALS TRANSPARENCY from the journal article. [Copy verbatim text and use quotation marks; do not otherwise edit the text (e.g., there is no need to remove line breaks or to edit characters that do not copy correctly). For multiple quotations, separate each using the word AND (capitalized).]

3) DESIGN AND ANALYSIS TRANSPARENCY

3a. Does the article indicate that the authors followed a reporting guideline?

[Reporting guidelines describe the minimum information about study methods and results that should be included in a journal article. Reporting guidelines differ from "style guides" that describe how information should be reported (rather than what information to report). Most reporting guidelines include a checklist and a flow diagram and have an acronym or name (e.g., "CONSORT" or "JARS"). There may not be an appropriate reporting guideline for some studies. First check the Abstract, then Methods, and then Results (you do not need to check the entire manuscript). Answer "Yes" if the article includes an explicit statement that they used a reporting guideline to develop the manuscript. Answer "No" if the article includes an explicit statement that they did not use a reporting guideline to develop the manuscript. Answer "No" if the study does NOT make reference to use of a reporting guideline. Answer "No" if the study includes a flow diagram but does NOT make explicit reference to use of a reporting guideline.]

Yes

No

3b. [Display IF 3a=Yes] Does the article include a completed form/checklist for the reporting guideline?

[Answer "Yes" if the article includes a link to or an appendix consisting of a completed checklist with a quotation or page number corresponding to each item in the reporting guideline.]

Yes

No

3c. Does the article indicate that the study protocol is publicly available?

[A study protocol is a document describing the study methods written before completing a study, which may be published separately or included in a final report (e.g., as an appendix). Answer "Yes" if the article includes an explicit statement indicating that a protocol or document that details the rationale, proposed methods, organization, and ethical considerations of a study is publicly available. Answer "Yes" if the report references or includes a standalone document that describes the methods for conducting the study and providing the intervention. Answer "No" if a study protocol is not included or methods for conducting the study or intervention are described only as part of a "Methods" section in a report. Answer "No" if the report states that there was not a study protocol. Answer "No" if the report states only that the protocol is available "upon request".)

Yes

No

3d. [Display IF 3c=Yes] Does the article include a link to or copy of the study protocol? [Answer "Yes, the article includes a reference to a published protocol (e.g., as

a journal article or a document on a website)” if the protocol is published as a document on a website other than a journal appendix/supplement. Answer “Yes, the article includes a DOI or permanent link to a trusted repository (e.g., ClinicalTrials.gov)” only if the article explicitly states that the PROTOCOL can be found at the repository/registry.]

Yes, the article includes a reference to a published protocol (e.g., as a journal article or a document on a website)

Yes, the article includes a DOI or permanent link to a trusted repository (e.g., ClinicalTrials.gov)

Yes, the article includes an appendix with the study protocol

No

3e. Directly copy and paste questions about DESIGN AND ANALYSIS TRANSPARENCY from the journal article. [Copy verbatim text and use quotation marks; do not otherwise edit the text (e.g., there is no need to remove line breaks or to edit characters that do not copy correctly). For multiple quotations, separate each using the word AND (capitalized).]

4) REGISTRATION OF STUDIES

4a. Does the article indicate whether the authors registered the study? [Answer “Yes, it states the study was registered” if the journal article includes a registration number or an explicit statement that the study has been registered. Answer “Yes, it states the study was NOT registered” if it states that the study was not registered. Answer “No” if the report makes no mention of study registration.]

Yes, it states the study was registered

Yes, it states the study was NOT registered

No

4b. [Display IF 4a=Yes, it states the study was registered] Does the article provide a study registration number or link to the time-stamped registration?
[Answer “Yes” if the article provides a study registration number (e.g., an NCT number for ClinicalTrials.gov) or a persistent identifier (e.g., a DOI) for the study registration.]

Yes

No

4c. [Display IF 4a=Yes, it states the study was registered] Does the article indicate the timing of the registration? [If the journal article uses a term to describe the timing of registration, select the term that most closely matches the term used in the journal article. For randomized trials, select “prospective” if the journal article indicates that the study was registered before beginning enrollment or within 21 days of beginning enrollment. For studies using existing data, select “prospective” if the journal article indicates that the study was registered before the authors had access to the data or conducted their analyses. Select “retrospective” if the journal article indicates that the study was registered after collecting the data or beginning analysis.]

Yes, prospective

Yes, retrospective

No, not reported

4d. [Display IF 4a=Yes, it states the study was registered] Does the article disclose whether changes were made to the registered plan (if any)? [Answer “Yes” if the article includes a statement about changes to the protocol, including that “no changes were made to the prospectively registered plan”. Answer “No” if no explicit statement is made.]

Yes, it states that changes were made

Yes, it states that changes were NOT made

No

4e. Directly copy and paste questions about REGISTRATION OF STUDIES from the journal article. [Copy verbatim text and use quotation marks; do not otherwise edit the text (e.g., there is no need to remove line breaks or to edit characters that do not copy correctly). For multiple quotations, separate each using the word AND (capitalized).]

5) REGISTRATION OF ANALYSIS PLANS

5a. Does the article indicate whether the authors registered the analysis plan? [An analysis plan is a standalone document or part of a study protocol that provides a detailed description of the planned data management and statistical methods. An analysis plan is written before completing a study. It may be included in a final report (e.g., as an appendix). Answer “Yes, it states the analysis plan was registered” if the journal article includes an explicit statement that the study has been registered. Answer “Yes, it states the analysis plan was NOT registered” if it states that the analysis plan was not registered. Answer “No” if the report makes no mention of an analysis plan and features of the statistical analysis are described only as part of a “Methods” section in a report.]

Yes, it states the analysis plan was registered

Yes, it states the analysis plan was NOT registered

No

5b. [Display IF 5a=Yes, it states the analysis plan was registered] Does the article provide instructions to locate or link to the registered analysis plan?
[Answer “Yes” if the article provides a registration number or identifier (e.g., an NCT number for ClinicalTrials.gov) and states that the analysis plan is included with the registration. Answer “Yes” if the article includes a persistent identifier (e.g., a DOI) for the analysis plan.]

Yes

No

5c. [Display IF 5a=Yes, it states the analysis plan was registered] Does the article indicate the timing of the analysis plan? [If the journal article uses a term to describe the timing of registration, select the term that most closely matches the term used in the journal article. For randomized trials, select “prospective” if the journal article indicates that the analysis plan was registered before beginning enrollment or within 21 days of beginning enrollment. For studies using existing data, select “prospective” if the journal article indicates that the study was registered before the authors conducted their analyses. Select “retrospective” if the journal article indicates that the study was registered after collecting the data or beginning analysis.]

Yes, prospective

Yes, retrospective

No, not reported

5d. [Display IF 5a=Yes, it states the analysis plan was registered] Does the article disclose whether changes were made to the registered analysis plan (if any)?

[Answer “Yes” if the article includes a statement about changes to the analysis plan, including that “no changes were made to the prospectively analysis plan”. Answer “No” if no explicit statement is made.]

Yes, it states that changes were made

Yes, it states that changes were NOT made

No

5e. [Display IF 5a=Yes, it states the analysis plan was NOT registered OR 5a=No] Does the article include a copy of or link to the analysis plan? [Answer “Yes” if the article includes a reference to an unregistered but published analysis plan (e.g., as a journal article or a document on a website). Answer “Yes” if the article includes an appendix with an unregistered analysis plan.]

Yes, the article includes a reference to a published analysis plan (e.g., as a journal article or a document on a website)

Yes, the article includes an appendix with the analysis plan

No

5f. Directly copy and paste questions about REGISTRATION OF ANALYSIS PLANS from the journal article. [Copy verbatim text and use quotation marks; do not otherwise edit the text (e.g., there is no need to remove line breaks or to edit characters that do not copy correctly). For multiple quotations, separate each using the word AND (capitalized).]

6) REPLICATION AND REGISTERED REPORTS

6a. Does the article include a statement about whether or not the study is a replication? [“Replication” studies aim to reproduce the methods and results of previous studies, and are typically described using the term “replication”. Answer “No” if the study states that it is replicating previous research/studies but does NOT indicate a specific previous study which they have attempted to replicate. Example: The aim of the present study was to perform an exact replication of the critical conditions and manipulations reported by Schiller et al. in their seminal study (Schiller et al., 2010, Experiment 1).]

Yes, the article states that the study is a replication

Yes, the article states that the study is NOT a replication

No

6b. Does the article include a statement that all outcomes have been reported, regardless of significance or novelty of findings? [Answer “Yes” if the article includes a transparency declaration affirming that this manuscript is an honest, accurate, comprehensive account of the study being reported (e.g., affirming that no outcomes have not been reported based on the nature of the findings). Answer "No" if the article mentions the word "bias" that is not rated to selective non-reporting of outcomes or analyses. Examples: 1. We report how we determined our sample size, all data exclusions, all manipulations, and all measures in the study. 2. The corresponding author affirms that this manuscript is an honest, accurate, and transparent account of the study being reported; that no important aspects of the study have been omitted; and that any discrepancies from the study as planned (and, if relevant, registered) have been explained.]

Yes

No

6c. Does the article indicate whether the study is a “Registered Report”? [First check the Abstract, then Methods, and then Objectives (you do not need to check the entire manuscript). Answer “Yes” if the article is labeled explicitly as a “Registered Report”. Answer “Yes” if the article states explicitly that the journal conducted a first stage of review prior to the data being collected (i.e., before the outcomes were observed) and made a preliminary decision about acceptance. Answer “No” if the study does NOT indicate explicitly that the study is a registered report.]

Yes

No

6d. [Display IF 6c=No] Does the article indicate whether the study underwent “results blind review”? [First check the Abstract, then Methods, and then Objectives (you do not need to check the entire manuscript). Answer “Yes” if the article refers explicitly to “result-blind” or “results-masked” review. Answer “Yes” if the article states

explicitly that the journal conducted a first stage of review based on the background and methods sections alone (i.e., without the results and discussion sections). Answer “No” if the study does NOT indicate explicitly that the study underwent results-blind review.]

Yes

No

6e. Directly copy and paste questions about REPLICATION AND REGISTERED REPORTS from the journal article. [Copy verbatim text and use quotation marks; do not otherwise edit the text (e.g., there is no need to remove line breaks or to edit characters that do not copy correctly). For multiple quotations, separate each using the word AND (capitalized).]

7) OPEN SCIENCE BADGES

7a. CHECK ALL THAT APPLY: Has the article been awarded any of the following open science badges? [“Open sciences badges” are digital badges for empirical articles that acknowledge the use of open science practices (i.e., preregistration, data sharing, and materials sharing) in the study report. Select any badges that are mentioned by name on the downloaded article or on the article located on the journal website. See: https://cos.io/our-services/open-science-badges/]

No, the article has not been awarded any open science badge
“Preregistration” Badge
“Open Data” Badge
“Open Materials” Badge

7b. Directly copy and paste questions about OPEN SCIENCE BADGES from the journal article. [Copy verbatim text and use quotation marks; do not otherwise edit the text (e.g., there is no need to remove line breaks or to edit characters that do not copy correctly). For multiple quotations, separate each using the word AND (capitalized).]
